# *Candida albicans* White and Opaque Cells Undergo Distinct Programs of Filamentous Growth

**DOI:** 10.1371/journal.ppat.1003210

**Published:** 2013-03-07

**Authors:** Haoyu Si, Aaron D. Hernday, Matthew P. Hirakawa, Alexander D. Johnson, Richard J. Bennett

**Affiliations:** 1 Department of Molecular Microbiology and Immunology, Brown University, Providence, Rhode Island, United States of America; 2 Department of Microbiology and Immunology, University of California, San Francisco, San Francisco, California, United States of America; Carnegie Mellon University, United States of America

## Abstract

The ability to switch between yeast and filamentous forms is central to *Candida albicans* biology. The yeast-hyphal transition is implicated in adherence, tissue invasion, biofilm formation, phagocyte escape, and pathogenesis. A second form of morphological plasticity in *C. albicans* involves epigenetic switching between white and opaque forms, and these two states exhibit marked differences in their ability to undergo filamentation. In particular, filamentous growth in white cells occurs in response to a number of environmental conditions, including serum, high temperature, neutral pH, and nutrient starvation, whereas none of these stimuli induce opaque filamentation. Significantly, however, we demonstrate that opaque cells can undergo efficient filamentation but do so in response to distinct environmental cues from those that elicit filamentous growth in white cells. Growth of opaque cells in several environments, including low phosphate medium and sorbitol medium, induced extensive filamentous growth, while white cells did not form filaments under these conditions. Furthermore, while white cell filamentation is often enhanced at elevated temperatures such as 37°C, opaque cell filamentation was optimal at 25°C and was inhibited by higher temperatures. Genetic dissection of the opaque filamentation pathway revealed overlapping regulation with the filamentous program in white cells, including key roles for the transcription factors *EFG1*, *UME6*, *NRG1* and *RFG1*. Gene expression profiles of filamentous white and opaque cells were also compared and revealed only limited overlap between these programs, although *UME6* was induced in both white and opaque cells consistent with its role as master regulator of filamentation. Taken together, these studies establish that a program of filamentation exists in opaque cells. Furthermore, this program regulates a distinct set of genes and is under different environmental controls from those operating in white cells.

## Author Summary

*Candida albicans* is the most common human fungal pathogen, capable of growing as a commensal organism or as an opportunistic pathogen. Perhaps the best-studied aspect of *C. albicans* biology is the transition between the single-celled yeast form and the multicellular filamentous form. This transition is necessary for virulence, as cells locked in either state are avirulent. Here, we demonstrate that the yeast-filament transition is tightly regulated by another morphological switch, the white-opaque phenotypic switch. White cells undergo filamentation in response to a wide range of established physiological cues, while opaque cells do not. We further show that opaque cells can indeed undergo filamentation, but that they do so in response to different environmental cues than those of white cells. We define the genetic regulation of filamentous growth in opaque cells, as well as the transcriptional profile of these cell types, and contrast them with the established program of filamentation in white cells. Our results reveal a close relationship between the white-opaque switch and the yeast-hyphal transition, and provide further evidence of the morphological plasticity of this pathogen. They also establish that epigenetic switching allows two fungal cell types with identical genomes to respond differently to environmental cues.

## Introduction

Morphological plasticity is key to the lifestyle of fungal pathogens such as *Candida albicans*, the most frequently isolated human fungal pathogen. The best-studied morphological switch in *C. albicans* is the transition between yeast and true hyphae or pseudohyphae (filamentous forms). Pseudohyphal cells are highly branched and consist of ellipsoidal cells with constrictions at the septa. In contrast, hyphal cells are less branched, have parallel sides, and lack constrictions at the septa [1], [2]. The yeast-hyphal switch regulates *C. albicans* pathogenesis, as hyphal forms adhere to and invade epithelial cells during mucosal infections, resulting in extensive damage to host cells [2]. The switch to hyphae is also induced upon phagocytosis by macrophages, allowing pathogen evasion from immune capture [3], [4]. Furthermore, the hyphal form is important for virulence in systemic models of disease, although it is not clear if the hyphal morphology *per se* or genes co-regulated with the morphological transition are critical for virulence [2].

The yeast-hyphal transition in *C. albicans* is induced in response to a wide variety of environmental stimuli including serum, neutral pH, nutrient limitation, high CO_2_ concentrations, and embedded conditions [2], [5]. The transcriptional regulation of filamentation is complex, but many stimuli act via two major signaling pathways: a cyclic AMP-dependent pathway that depends on the Efg1 transcription factor, and a mitogen-activated protein kinase (MAPK) pathway that activates the Cph1 transcription factor [6], [7]. In addition, most filamentation-inducing conditions require a temperature of 37°C (or higher) for efficient filamentous growth [2], [5]. The temperature requirement appears to be mediated by Hsp90, as compromising Hsp90 activity promotes filamentation in response to serum at 30°C [8].

A second morphological switch involves the interconversion between white and opaque forms of *C. albicans*. This is an epigenetic switch that allows rapid and reversible switching between white cells that are round and opaque cells that are ellipsoidal [9]. Genes present at the *Mating-Type Like* (*MTL*) locus strictly regulate the white-opaque switch, so that only *MTL* **a** or *MTL*α strains can undergo the transition to the opaque form, while *MTL* **a**/α strains are permanently locked in the white state [10]. Opaque cells are the mating-competent form of *C. albicans*, undergoing mating approximately a million times more efficiently than white cells [10]. In addition to mating, the white-opaque switch also regulates multiple other facets of *C. albicans* biology. White cells are more virulent in systemic infections than opaque cells, while conversely opaque cells are better colonizers in skin infections than white cells [11], [12]. The two cell states also interact differently with immune cells, as opaque cells are less susceptible to phagocytosis by macrophages [13]. It is therefore apparent that white and opaque cells are differentially programmed in many aspects of their behavior, including their interaction with the host.

In this work, we compare the ability of the two phenotypic states, white and opaque, to undergo filamentation. Studies on filamentation have almost exclusively utilized cells in the white state. In this form, cells are readily induced to form hyphae or pseudohyphae in response to diverse stimuli. In contrast, opaque cells do not undergo filamentation in response to these stimuli and typically grow only in the budding yeast form [14]. One indication that opaques may form filaments under specialized conditions came from studies using a perfusion chamber; opaque cells attached to the wall of the chamber formed hyphae, but did not do so when grown in suspension [15]. Additionally, opaque cells can undergo polarized growth in response to mating pheromones, establishing that these cell types are competent for filament-like growth [16]–[18].

Here, we establish that opaque cells fail to form filaments under a variety of environmental conditions that induce efficient hyphal growth in white cells including serum, neutral pH, and nutrient limitation. In contrast, we show that opaque cells undergo efficient filamentation under certain conditions in liquid and solid media, but these conditions are different from those that induce filamentation of white cells. In particular, growth of opaque cells in certain nutrient-poor media or in the presence of the sugar sorbitol efficiently induced filamentous growth, whereas white cells continued to grow as budding yeast under these conditions. Both hyphal-like and pseudohyphal-like cells were observed in opaque cells, but the ratio depended on the inducing signal. In addition, thermal regulation of filamentation in opaque cells is reversed relative to that in white cells. While filamentation in white cells is optimal at 37°C under most conditions, opaque cells underwent filamentation efficiently at 25°C, and elevated temperatures suppressed filamentous growth. Genetic analysis and transcriptional profiling of filamentous opaque cells revealed both similarities and differences between the filamentation programs in opaque and white cells. These studies establish nutrient-controlled filamentous growth in *C. albicans* opaque cells and indicate fundamental differences between the environmental cues regulating filamentation in white and opaque cells.

## Materials and Methods

### Media and reagents

Synthetic complete dextrose medium (SCD) and yeast extract peptone dextrose medium (YPD) were made as described previously [19]. YPD plates containing 200 µg/ml nourseothricin were used for selection of strains that were resistant to nourseothricin (Werner Bioagents, Jena, Germany) as previously described [20]. SCD low phosphate (LP) medium was made with yeast nutrient base (YNB) w/o phosphate (cat. #CYN6701, Formedium LTD, Hunstanton, England) and KH_2_PO_4_ was supplemented to a final concentration of 10 µM. LP medium was adjusted to pH 4.7 with 3 M HCl before autoclaving. Synthetic low ammonium dextrose (SLAD) medium was made as described previously [21]; 2% agar was washed five times with distilled water, autoclaved with 1.7 g/L YNB w/o ammonia, and then supplemented with ammonium sulfate and dextrose to final concentrations of 50 µM and 2%, respectively. Sorbitol medium (SOR) was SCD supplemented with 1 M sorbitol. Minimal (MIN) medium consisted of 7 g/L YNB and 2% dextrose. Spider medium contained 1.35% agar, 1% nutrient broth, 0.4% potassium phosphate, and 2% mannitol (pH 7.2). N-acetyl glucosamine containing medium (GlcNAc) was modified Lee's medium [22] without glucose but supplemented with 1.25% N-acetyl glucosamine (Sigma). Neutral pH medium was made by buffering SCD with 150 mM HEPES (pH 7.0). Calcofluor white stain was obtained from Fluka Biochemika and geldanamycin from A.G. Scientific, Inc.

### Plasmids and strains

*C. albicans* strains used in this study are listed in Table 1 and oligonucleotides in Table S1. All strains are derived from SC5314 unless stated otherwise. To construct the *pACT1-WOR1* plasmid, the promoter of the *ACT1* gene was PCR amplified with oligonucleotides 610 and 611 and the *WOR1* ORF with oligonucleotides 2421 and 2422. The two fragments were then combined in a single fusion PCR reaction using oligos 610 and 2422 and cloned into plasmid pSFS2A [20] between *Apa*I and *Xho*I restriction sites. A modified pSFS2A plasmid was also constructed in which the *SAT1* gene was replaced with the gene for hygromycin B resistance. A region of plasmid pSFS2A was PCR amplified with oligos 1669 and 1652 and the gene for hygromycin B resistance amplified from plasmid pYM70 [23] with oligos 1651/1653. The two PCR products were combined by fusion PCR using oligos 1669/1651 and cloned between *Hin*dIII and *Pst*I restriction sites in the pSFS2A backbone. The resulting *pACT1-WOR1* plasmids (either with *SAT1* or *HYG* markers) were digested with *Bgl*II to linearize in the *pACT1* region and integrated into the endogenous *ACT1* locus to obtain constitutive *WOR1* expression. Correct integration was confirmed by PCR.

**Table 1:** Strains used in this study.

Strains	Genotype	Mating Type	Source
RBY717	*MTL* ***a*** */MTL* ***a*** * (white)	**a**/**a**	[67]
RBY731	*MTL* ***a*** */MTL* ***a*** * (opaque)	**a**/**a**	[67]
CAY1550	*MTL* ***a*** */MTL*αΔ*::FRT tup1::hisG/tup1::p405-URA3 ura3/ura3* (white)	**a**/Δα	Derived from BCa2-10 [41]
CAY1552	*MTL* ***a*** */MTL*αΔ*::FRT nrg1::hisG/nrg1::hisG-URA3-hisG ura3/ura3* (white)	**a**/Δα	Derived from BCa23-3 [46]
CAY1571	*ume6::LEU2/ume6::HIS1 arg4::ARG4/arg4 leu2/leu2 his1/his1 pAct1-WOR1::SAT1-FLIP* * (opaque)	α/α	Derived from DK312 [25]
CAY1616	*MTL* ***a*** */MTL*αΔ*::FRT tup1::hisG/tup1::p405-URA3 ura3/ura3 pAct1-WOR1::SAT1-FLIP* (opaque)	**a**/Δα	Derived from BCa2-10 [41]
CAY1618	*MTL* ***a*** */MTL*αΔ*::FRT nrg1::hisG/nrg1::hisG-URA3-hisG ura3/ura3 pAct1-WOR1::SAT1-FLIP* (opaque)	**a**/Δα	Derived from BCa23-3 [46]
CAY2091	*MTL* ***a*** */MTL*αΔ*::SAT1-FLIP cph2::LEU2/cph2::HIS1 arg4/arg4 leu2/leu2 his1/his1* * (white)	**a**/Δα	This study
CAY2214	*rfg1::LEU2/rfg1::FRT leu2/leu2 his1/his1 arg4/arg4* * (white)	**a**/**a**	This study
CAY2646	*tec1*Δ*::FRT/tec1*Δ*::FRT* * (white)	**a**/**a**	This study
CAY2688	*tec1*Δ*::FRT/tec1*Δ*::FRT* * (opaque)	**a**/**a**	This study
CAY2723	*ras1::LEU2/ras1::ARG4 leu2/leu2 his1/his1 arg4/arg4 gal1/gal1* * (white)	**a**/**a**	This study
CAY2795	*ras1::LEU2/ras1::ARG4 leu2/leu2 his1/his1arg4/arg4 gal1/gal1* * (opaque)	**a**/**a**	This study
CAY2701	*rfg1::LEU2/rfg1::FRT efg1::HIS1/efg1::ARG4 leu2/leu2 his1/his1 arg4/arg4* * (white)	**a**/**a**	This study
CAY2822	*rfg1::LEU2/rfg1::FRT efg1::HIS1/efg1::ARG4 leu2/leu2 his1/his1 arg4/arg4 pAct1-WOR1::SAT1* * (opaque)	**a**/**a**	This study
CAY2903	*MTL* ***a*** */MTL* ***a*** * pAct1-WOR1::SAT1-FLIP* * (opaque)	**a**/**a**	This study
CAY3151	*rfg1::LEU2/rfg1::FRT cph1::HIS1/cph1::ARG4 leu2/leu2 his1/his1 arg4/arg4 pAct1-WOR1::SAT1* * (opaque)	**a**/**a**	This study
CAY3292	*MTL* ***a*** */MTL*αΔ*::FRT efg1::LEU2/efg1::HIS1 arg4::ARG4/arg4 leu2/leu2 his1/his1 pAct1-WOR1::SAT1* * (opaque)	**a**/Δα	This study
CAY3294	*MTL* ***a*** */MTL*αΔ*::FRT czf1::LEU2/czf1::HIS1 arg4::ARG4/arg4 leu2/leu2 his1/his1 pAct1-WOR1::SAT1* * (opaque)	**a**/Δα	This study
CAY3296	*MTL* ***a*** */MTL*αΔ*::FRT cph2::LEU2/cph2::HIS1 arg4::ARG4/arg4 leu2/leu2 his1/his1 pAct1-WOR1::SAT1* * (opaque)	**a**/Δα	This study
CAY3298	*cph1::LEU/cph1::HIS1 arg4::ARG4/arg4 leu2/leu2 his1/his1 pAct1-WOR1::SAT1* * (opaque)	**a**/**a**	This study
CAY3299	*rfg1::LEU2/rfg1::FRT his1::HIS1/his1 arg4::ARG4/arg4 leu2/leu2 pAct1-WOR1::SAT1* * (opaque)	**a**/**a**	This study
CAY3522	*MTL* ***a*** */MTL*αΔ*::SAT1-FLIP czf1::LEU2/czf1::HIS1 leu2/leu2 his1/his1 arg4::ARG4/arg4* * (white)	**a**/Δα	This study
CAY3524	*cph1::LEU2/cph1::HIS1 arg4::ARG4/arg4 leu2/leu2 his1/his1* * (white)	**a**/**a**	This study
CAY3526	*efg1::LEU2/efg1::HIS1 arg4::ARG4/arg4 leu2/leu2 his1/his1 MTL* ***a*** */MTL*αΔ*::SAT1-FLIP* * (white)	**a**/Δα	This study
CAY3528	*rfg1::LEU2/rfg1::SAT1 leu2/leu2 his1::HIS1/his1 arg4/arg4* * (white)	**a**/**a**	This study
CAY3619	*pRAS1::GFP::RAS1* (opaque)	**a**/**a**	This study
CAY3621	*pRAS1::GFP::RAS1^G13V^* (opaque)	**a**/**a**	This study
CAY3697	*ume6::LEU2/ume6::HIS1/UME6::HYG arg4::ARG4/arg4 leu2/leu2 his1/his1 pAct1-WOR1::SAT1-FLIP* * (opaque)	α/α	This study
CAY3749	*mtla1::HisG*/*MTL*α *ura3/ura3 pRAS1::GFP::RAS1* (white)	Δ**a**/α	Derived from CHY257 [10]
CAY3751	*MTLa/mtl*α*1::HisG mtlα2::HisG ura3/ura3 pRAS1::GFP::RAS1^G13V^* (white)	**a**/Δα	Derived from CHY439 [10]
CAY4193	*hgc1*Δ*::FRT/hgc1*Δ*::FRT* # (opaque)	**a**/**a**	This study
CAY4197	*hgc1*Δ*::FRT/hgc1*Δ*::FRT::HGC1::SAT1* # (opaque)	**a**/**a**	This study
CAY4291	*MTLa/MTL*αΔ::*FRT tup1::hisG/tup1::p405-URA3 pAct1-WOR1::HYG pOP4-GFP::SAT1* (opaque)	**a**/Δα	This study
CAY4353	*MTLa/MTL*αΔ*::FRT tup1::hisG/tup1::p405-URA3 pOP4-GFP::SAT1* (white)	**a**/Δα	This study
CAY4356	*MTLa/MTL*αΔ*::FRT tup1::hisG/tup1::p405-URA3 pWH11-mCherry::SAT1* (white)	**a**/Δα	This study
CAY4479	*MTLa/MTL*αΔ*::SAT1 cph1::LEU2/cph1::HIS1 leu2/leu2 his1/his1 arg4/arg4* * (white)	**a**/Δα	This study
CAY4384	*MTL* ***a*** */MTL*αΔ*::FRT efg1::LEU2/efg1::HIS1/EFG1::HYG leu2/leu2 his1/his1 arg4::ARG4/arg4 pAct1-WOR1::SAT1* * (opaque)	**a**/Δα	This study
CAY4492	*MTLa/MTL*αΔ*::FRT tup1::hisG/tup1::p405-URA3 pAct1-WOR1::HYG pWH11*-*mCherry::SAT1* (opaque)	**a**/Δα	This study
CAY4502	*MTLa*Δ*::HYG/MTL*α*; HIS1/his1::FRT tetR UME6/FLP-CaNAT1 tetO-UME6* (opaque)	Δ**a**/α	Derived from MBY208 [68]
CAY4504	*HIS1/his1::FRT tetR UME6/FLP-CaNAT1 tetO-UME6* (white)	Δ**a**/α	Derived from MBY208 [68]
CAY4522	*efg1::LEU2/efg1::HIS1 arg4/arg4 leu2/leu2 his1/his1* * (white)	**a**/α	[26]
CJN2471	*cph1::LEU2/cph1::HIS1 leu2/leu2 his1/his1 arg4/arg4* * (white)	**a**/α	Gift of C. Nobile
DK340	*ume6::LEU2/ume6::HIS1 arg4::ARG4/arg4 leu2/leu2 his1/his1* * (white)	α/α	Derived from DK312 [25]

* strains also contain the genotype *ura3::imm434/URA3 iro1::imm434/IRO1*.

# *hgc1* mutant strains constructed in the P37005 strain background of *C. albicans*.

To target *RFG1* for deletion, a fusion PCR product was created as described previously [24]. Briefly, oligos 984/992 and 985/993 were used to PCR the 5′ and 3′ homologous flanks of *RFG1* and these flanks combined with a selectable marker (*LEU2*) by fusion PCR [24]. The pSFS2 (*rfg1::SAT1* flipper) plasmid [25] (a gift from David Kadosh, University of Texas San Antonio) was used to delete the second copy of *RFG1* and generate *rfg1::LEU2/rfg1::SAT1* double deletion mutants, as previously described [25]. The *SAT1* marker was subsequently excised by growth on maltose medium [20] to obtain strain CAY2214. Fusion PCR was also used to delete *CPH1* or *EFG1* from CAY2214, using the selectable markers *HIS1* and *ARG4* to generate *rfg1/cph1* and *rfg1/efg1* double mutants. Oligos 982/990 and 983/991 were used to PCR amplify the 5′ and 3′ flanks of the *CPH1* gene, and oligos 1215/2425 and 1216/1217 used to amplify the 5′ and 3′ flanks of the *EFG1* gene. The *MTL*α locus was also deleted from *efg1*, *czf1* and *cph2* mutant strains acquired from the Fungal Genetics Stock Center (strains originally generated by Homann *et al.* [26]) using the plasmid pRB102, as previously described [27]. The *SAT1* marker was subsequently excised by growth on maltose medium [20] to generate *MTL* **a** */MTL*αΔ*::FRT* strains. Correct integration of constructs was verified by PCR across 5′ and 3′ disruption junctions, and loss of the ORF was confirmed with primers internal to the open reading frame. Auxotrophic strains were also transformed with *C. albicans LEU2*, *HIS1*, or *ARG4*, PCR amplified by oligos 2490/2491, 2492/2493 or 2494/2495, respectively, to restore prototrophy to these strains.

White- and opaque-specific reporter constructs were generated as follows. For the opaque-reporter, the *SAT1* gene was PCR amplified from pSFS2A [20] using primers 169/170 and cloned into pCR-Blunt II-TOPO (Invitrogen) between *Xho*I and *Xba*I restriction sites. A triple mCherry reporter was next integrated into this vector by PCR amplifying three copies of the mCherry gene from plasmid pADH77 [28] with oligonucleotides 1846/1847, 1848/1849 and 1850/1386. The three mCherry PCR products were stitched together using *Bam*HI/*Afl*II, *Afl*II/*Stu*I, and *Stu*I/*Sal*I restriction sites and integrated between *Bam*HI and *Xho*I sites in the vector backbone to generate plasmid pRB224. The *C. albicans OP4* promoter was then PCR amplified using oligos 1974/1975 and cloned between *Kpn*I and *Sac*I restriction sites in pRB224 to generate the final reporter construct pRB227. This plasmid was linearized in the *OP4* gene with *Bsg*I and transformed into *C. albicans* as an opaque-specific reporter. For the white-cell reporter, the *C. albicans WH11* gene promoter was PCR amplified with oligos 1384/1396 and the GFP gene amplified from pADH76 [28] with oligos 1385/1386. These PCR products were fused by PCR with oligos 1384 and 1386, digested with *Apa*I/*Sal*I and cloned into pSFS2A to generate pRB168. This plasmid was linearized within the *WH11* gene with *Aat*II and transformed into *C. albicans* to generate a white-specific reporter.

An *EFG1* complementation plasmid was constructed by PCR amplification of *EFG1* using oligos 1838/1839, and cloning between *Apa*I and *Kpn*I sites in the modified pSFS2A for hygromycin B resistance. The resulting *EFG1* addback plasmid pRB326 was linearized by *Hpa*I and integrated into the endogenous *EFG1* locus in the *efg1* mutant strain CAY3292. Similarly, oligos 1832/1833 were used to PCR amplify *UME6* and the PCR product cloned between *Apa*I and *Kpn*I sites in pSFS2A (hygromycin B) to generate plasmid pRB328. This plasmid was linearized by *Sma*I and integrated into the endogenous *UME6* locus in the opaque *ume6* mutant strain CAY1571 to obtain strain CAY3697.

Construction of a *UME6* overexpressing strain was achieved using derivatives of strain MBY208, a gift of David Kadosh. MBY208 contains a construct expressing high levels of an *Escherichia coli tet* repressor-*Saccharomyces cerevisiae* Hap4 activation domain fusion protein, as well as a second construct expressing the *UME6* gene under the control of the *E. coli tet* operator [29]. The *MTL*α locus was deleted in MBY208 using a derivative of pRB102 (contains hygromycin B marker) to generate white (CAY4504) and opaque (CAY4502) strains.

### RNA sample preparation

Single opaque or white colonies were inoculated into liquid LP and SOR media at room temperature. Cells were harvested by centrifugation after 12 hours (LP) or 16 hours (SOR), and pellets frozen in liquid nitrogen. Total RNA was extracted from cell pellets (8–10 OD) following the RiboPure-Yeast Kit protocol (Applied Biosystems, Bedford, MA). RNA was treated with DNaseI (Applied Biosystems) to eliminate DNA contamination and re-extracted with phenol/chloroform. For quality control, RNA was analyzed using an Agilent 2100 Bioanalyzer to check RNA integrity.

### Hybridization of cDNA to microarrays and data analysis

Aminoallyl-labeled cDNA synthesis and hybridization to microarrays was previously described by Tuch *et al.* [30]. Arrays were scanned on a GenePix 4000 scanner (Axon Instruments), data quantified using GENEPIX PRO version 3.0 and normalized using Goulphar (http://transcriptome.ens.fr/goulphar). Pairwise average linkage clustering analysis was performed using CLUSTER and visualized by TREEVIEW [31]. Significance Analysis of Microarrays (SAM, http://www-stat.stanford.edu/~tibs/SAM/) [32] and R (Ver 2.15.2, http://www.r-project.org/) were used to screen the statistically significant genes induced in filamentous opaque cells in LP or SOR medium versus SCD medium (four replicas each). The parameters used for screening and SAM results are provided in the supplemental data (see tables in Text S1 and Text S2). The Candida genome database (www.candidagenome.org) and the Yeast Genome Database (http://www.yeastgenome.org/) were used to facilitate further analysis. Array data has been uploaded to GEO (accession number GSE42963).

### Sample preparation and light microscopy

White and opaque cells were streaked onto thin agar plates. After 22 hours of growth, a small square (∼1 cm^2^) was cut from the plate and stained with calcofluor white. Digital images of cells were collected with Infinity analyzer software and an Infinity 2 digital camera (Lumenera Corporation, Ottawa, Canada). DIC and fluorescent images were collected with a Zeiss Inverted Microscope (Axio Observer. Z1) fitted with an AxioCam HR. Images were processed with AxioVision Rel. 4.8 (Zeiss, Germany).

### Scanning electron microscopy

*C. albicans* opaque cells were grown on solid SOR or LP media at 25°C for 22 hours. *C. albicans* white cells were grown in liquid YPD + 10% serum at 37°C for 2 hours. Cells were resuspended in water and attached to poly-L-lysine coated-coverslips. Cells were fixed with 2.5% (w/v) glutaraldehyde in 0.1 M Na-cacodylate buffer, pH 7.4 at 4°C, and washed with 0.1 M Na-cacodylate buffer, pH 7.4. The cells were postfixed with 1% aqueous osmium tetroxide in 0.1 M Na-cacodylate buffer, pH 7.4 at 25°C for 90 minutes, and washed with 0.1 M Na-cacodylate buffer, pH 7.4. Following fixation, cells were dehydrated gradually using a gradient ethanol series and subsequently dried in a critical point dryer. The samples were then coated with 20 nm gold palladium (60∶40) in an Emitech K550 sputter coater. Cells were imaged with a Hitachi S-2700 scanning electronic microscopy and collected with Quartz PCI software.

## Results

### Opaque cells do not undergo filamentation under conditions that induce hyphal growth in white cells

**Figure 1:**
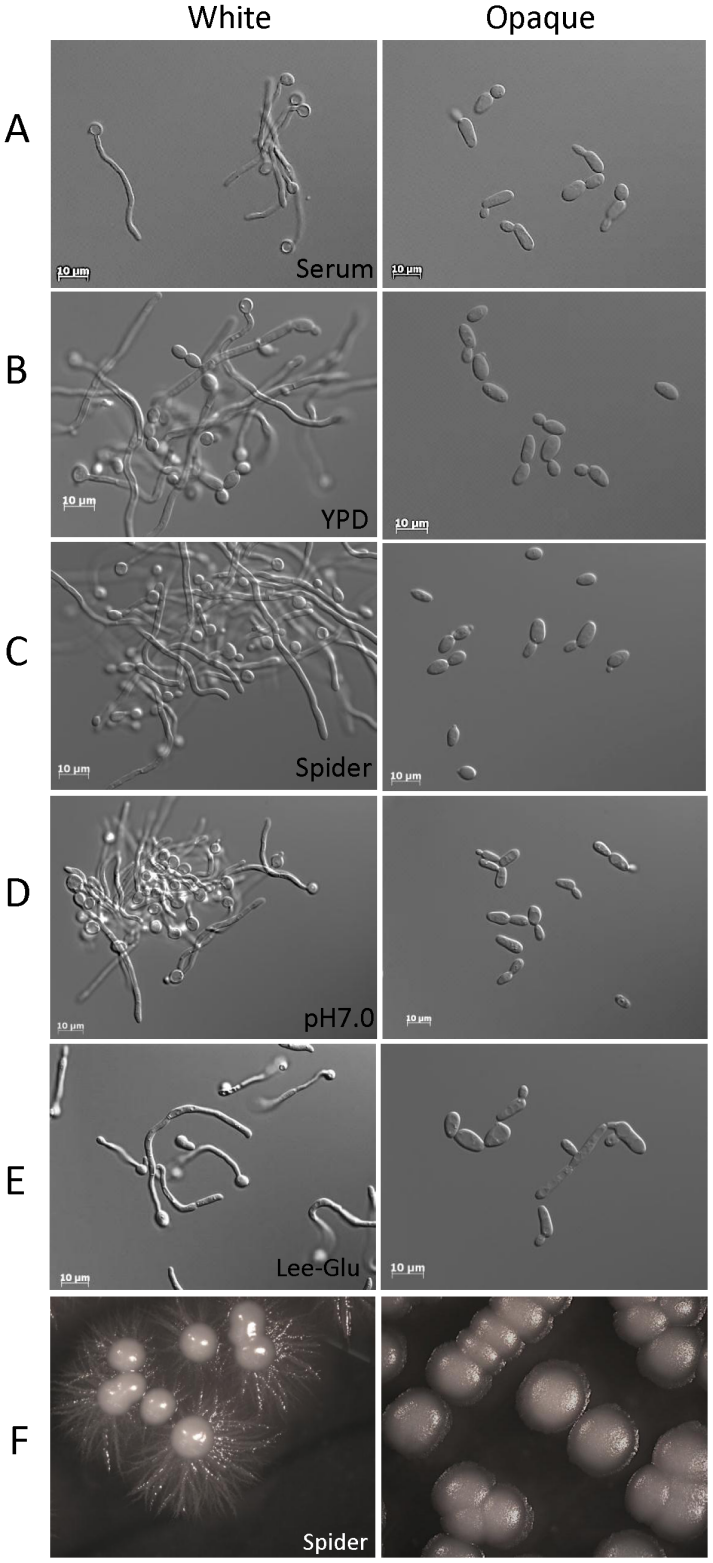
Conditions inducing white cell filamentation do not induce opaque cell filamentation. Environmental cues that induce efficient filamentation in *C. albicans* white cells (RBY717) include serum (A), high temperature (B), Spider medium (C and F), neutral pH medium (D), and Lee's medium (E). None of these environmental cues induce filamentous growth in opaque cells. Cell photographs were taken after 5 hours incubation at 37°C except for YPD supplemented with serum (2 hour incubation). Colonies grown on Spider medium were incubated at 30°C for 4 days.

Filamentation of *C. albicans* white cells occurs in response to a wide variety of environmental stimuli, including neutral pH, serum, nutrient limitation, and CO_2_ [2], [5]. We first addressed whether *C. albicans* opaque cells can undergo filamentous growth under any of the established conditions that promote white cell filamentation. As opaque cells are unstable at 37°C *in vitro* [9], *MTL* **a**/**a** strains were locked in the opaque state by expressing the *WOR1* gene under a constitutive promoter. *WOR1* is the master regulator of the white-opaque switch, and constitutive expression of *WOR1* ensures that cells are stably maintained in the opaque state [33]–[35]. As shown in Figure 1, white cells efficiently formed hyphae when grown in YPD medium supplemented with serum at 37°C, in low-nutrient Spider medium at 30° or 37°C, or in neutral pH Lee's medium at 37°C. Growth in YPD medium at 37°C was also sufficient to induce filamentous growth in white cells, as previously observed [36] (Figure 1B). In contrast, opaque cells did not undergo efficient filamentation under any of these culture conditions, and instead continued to grow predominantly as budding yeast cells (Figure 1). For example, > 99% of white cells grown in YPD+serum medium underwent filamentation, while less than 5% of opaque cells formed filaments under the same conditions. These results establish that *C. albicans* opaque cells do not form filaments efficiently under many of the standard *in vitro* conditions that induce white cell filamentation.

### Opaque cells undergo efficient filamentation in response to unique environmental cues

We next screened a series of *in vitro* culture conditions to identify environments that induce robust filamentous growth in opaque cells. Opaque-locked strains (overexpressing *WOR1*) or natural opaque strains were cultured on a variety of media, and colony and cell morphologies examined for evidence of filamentation. At least three distinct environmental cues were found to activate a program of opaque cell filamentation.

**Figure 2:**
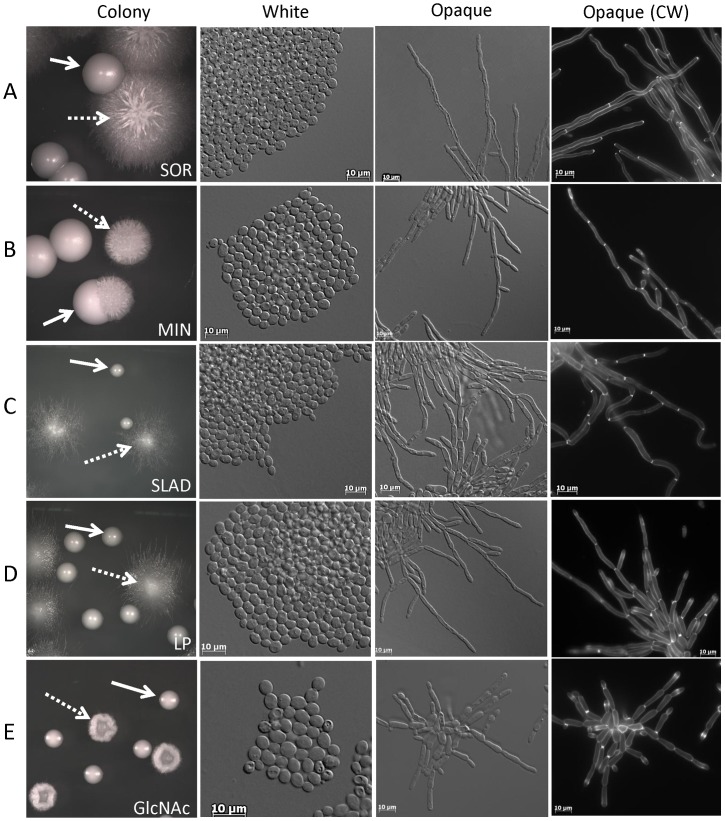
Novel environmental cues induce filamentation in *C. albicans* opaque cells. Several culture conditions are described that induce filamentous growth in opaque-locked **a** cells (CAY2903). These include growth on (A) sorbitol (SOR), (B) minimal (MIN), (C) low nitrogen (SLAD), (D) low phosphate (LP) or (E) N-acetyl glucosamine (GlcNAc) medium at 25°C. These culture conditions do not induce filamentation in white **a** cells (RBY717). Panels show colony morphologies from mixed white/opaque populations (solid arrow, white colony; dashed arrow, opaque colony). Additional panels show white cells, opaque cells, and calcofluor white (CW)-strained opaque cells.

First, growth of opaque cells on medium containing the sugar sorbitol (synthetic complete dextrose medium supplemented with 1 M sorbitol; SOR medium) produced highly wrinkled colonies containing cells that were highly filamentous (Figure 2A). Filamentation was induced within 24 hours, and colony and cell morphologies are shown at 4 days of growth at 25°C. Staining of the cell walls with calcofluor white revealed that filaments consisted of cells with parallel sides and no constrictions at the septa, similar to true hyphae formed by white cells (Figure 2A).

Second, growth on minimal medium (MIN medium; synthetic medium which lacks amino acids) induced filamentous growth producing highly wrinkled colonies (Figure 2B). Colony phenotypes were even more marked in minimal medium lacking nitrogen (SLAD medium) or minimal medium containing low phosphate concentrations (LP medium), as these conditions induced extensive peripheral filamentation around the edges of the colonies (Figure 2C and D). Filamentation was induced within 24 hours and colonies are shown after 4 days of growth at 25°C. Filaments from MIN, SLAD, and LP resembled pseudohyphae rather than true hyphae, as cells were highly elongated and branched, with slight constrictions at each of the branch points (Figure 2B–D).

Third, growth of opaque cells in the presence of the carbon source N-acetyl glucosamine (GlcNAc medium; Lee's medium containing 1.25% GlcNAc) induced efficient filamentation. Filamenting opaque cells resembled pseudohyphal cells with constrictions present at the septa (Figure 2E, cells shown after 24 hours and colonies after 4 days at 25°C). GlcNAc and SLAD have previously been reported to be activators of hyphal growth in white cells [37], [38], but we found that this required extended incubation for 5 days or longer at 37°C. In fact, white cells did not undergo filamentation under any of the conditions that efficiently induce opaque cell filamentation (compare white and opaque cell and colony morphologies in Figure 2).

Filamentous phenotypes were similar when using either natural opaque cells or those locked into the opaque state by constitutive *WOR1* expression (compare Figure 2 and Figure S1). In addition, removal of cells from the filamenting opaque colonies gave rise to regular opaque colonies when incubated on SCD medium (data not shown). This result indicates that opaque cells did not switch to the white state when grown under filament-inducing conditions but were stably maintained as opaque cells.

The results described above were achieved using culture on solid media, but filamentation was also observed in liquid culture. For example, growth of opaque cells in liquid SOR or LP medium was found to efficiently induce filamentous growth (Figure S2B and C). In contrast filamentation in MIN or SLAD media was considerably reduced compared to that on solid media (Figure S2D and E). We therefore establish that opaque cells can filament in response to different environmental cues, and can do so when grown both in liquid culture and on agar plates.

**Figure 3:**
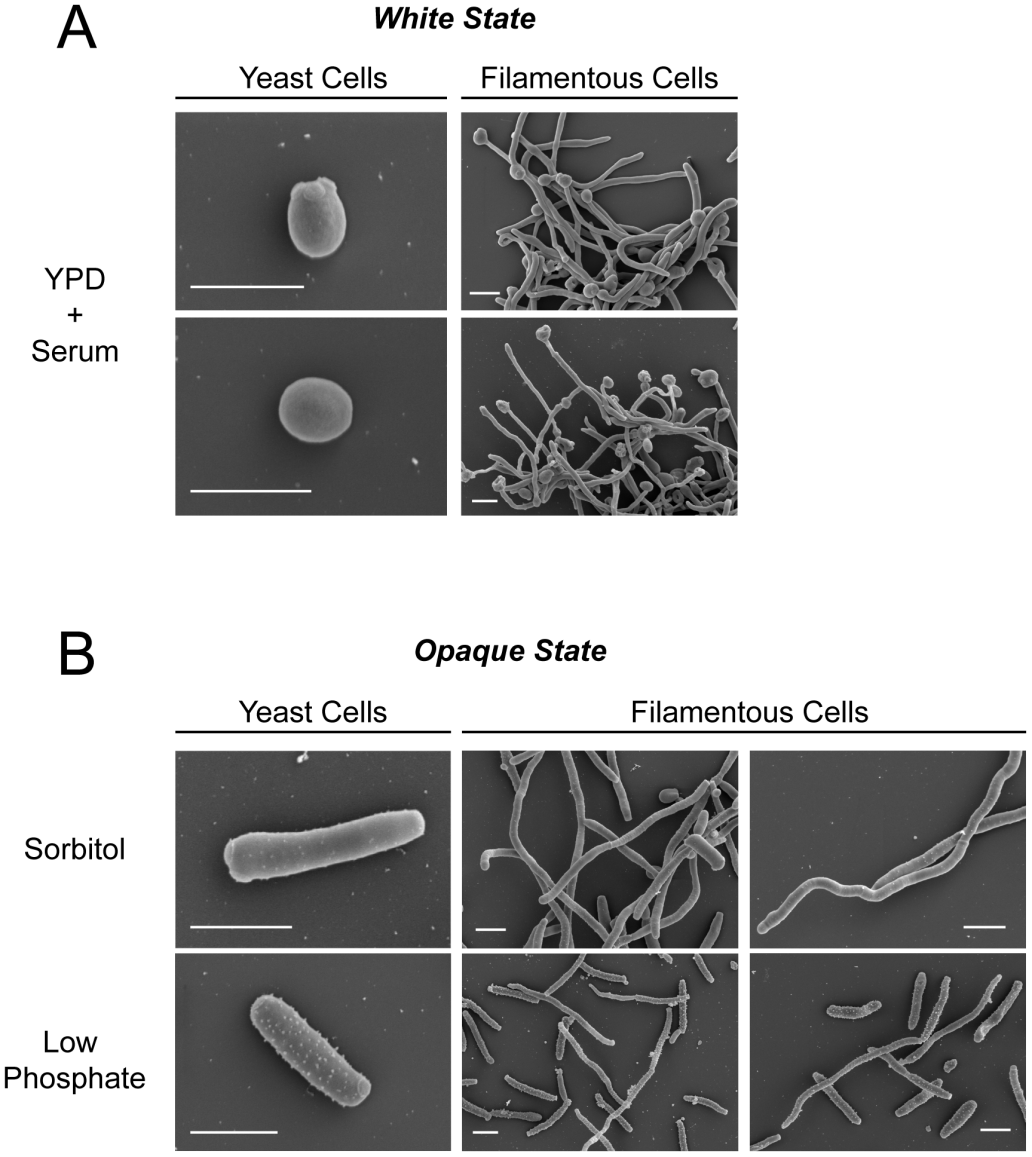
Scanning electron micrographs of yeast and filamentous forms of *C. albicans* white and opaque cells. (A) White cells (RBY717) were induced to form hyphae by growth in YPD supplemented with serum at 37°C. (B) Opaque cells (CAY2903) were induced to undergo filamentous growth by culture on LP or SOR media at 25°C. Scale bar, 5 µm.

The structure of filamentous opaque cells was further analyzed by scanning electron microscopy and compared to that of hyphal white cells (Figure 3). These images confirmed that opaque cells grown on SOR medium resembled hyphal cells (parallel sides with no constrictions) while those grown on LP medium resembled pseudohyphal cells (constrictions between elongated buds). These results establish that opaque cells can undergo a program of filamentous growth including either pseudohyphal-like or hyphal-like cells. Furthermore, we show that the environmental cues regulating filamentation in white and opaque cells are distinct, with different cues inducing filamentous growth in the two phenotypic states.

### Differential regulation of white/opaque filamentation by temperature

**Figure 4:**
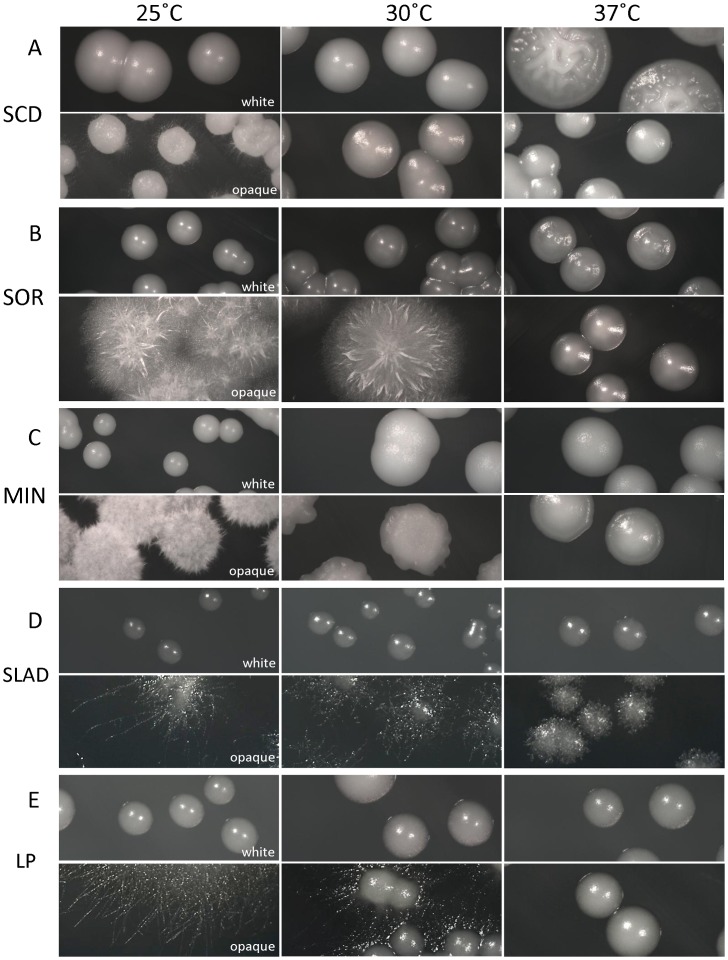
Differential thermal regulation of filamentous growth in white and opaque cells. White (RBY717) and opaque (CAY2903) cells were cultured on SCD or filamentation-inducing media (SOR, MIN, SLAD, or LP) at 25°, 30°, or 37°C for 4 days. Opaque filamentation was optimal in the order 25°C > 30°C > 37°C. In contrast, white cell filamentation increased as temperatures increased, as evidenced by weak filamentation of white colonies on SCD medium at 37°C (top panels). (A) SCD, (B) SOR, (C), MIN, (D), SLAD, or (E) LP medium.

The yeast-hyphal switch in *C. albicans* white cells is strongly influenced by temperature, with most filamentation-inducing conditions occurring at elevated temperatures (e.g. serum induction of hyphae is increased at 37°C compared to 30°C [8]), although white cells also undergo filamentation at 25°C in response to certain cues [39], [40]. To determine if opaque cell filamentation is temperature-dependent, filament-inducing conditions were tested at 25°C, 30°C, and 37°C. Opaque filamentation was most efficient at 25°C, with decreased filamentation observed at 30°C, and even less filamentation at 37°C (Figure 4). We confirmed that the lack of filamentation at 37°C was not due to opaque cells switching back to white by using opaque-locked strains that have constitutive *WOR1* expression. The thermal regulation of filamentation was similar when compared for SOR, MIN, SLAD and LP media, indicating that elevated temperatures generally inhibit opaque cell filamentation (Figure 4B–E). Again, white cells did not filament efficiently under any of the tested media conditions even when incubated at 37°C (Figure 4A–E), further demonstrating the specificity of the program of opaque filamentation.

The heat shock protein Hsp90 has been implicated as a key regulator of temperature sensing in *C. albicans*. Elevated temperatures compromise Hsp90's functional capacity and this is thought to promote filamentation of white cells [8]. Pharmacological inhibition of Hsp90 (e.g. using the drug geldanamycin) therefore stimulates filamentation in white cells [8]. We used geldanamycin (GdA) to test if loss of Hsp90 also affects filamentation in opaque cells. White and opaque cultures were treated with geldanamycin in liquid YPD medium at 25°C and 30°C. Inhibition of Hsp90 caused efficient induction of filamentation in white cells at 30°C, but only a very limited morphological response in opaque cells (Figure S3). In fact, opaque cells were more susceptible to geldanamycin treatment than white cells, with the drug causing cell death in a large proportion of the population. Surviving opaque cells exhibited mixed morphologies, indicating that Hsp90 does not play as dominant a role in regulating opaque cell filamentation as it does in white cells.

### The role of MAPK and cAMP signal transduction pathways in opaque filamentation

Multiple signaling pathways regulate filamentation in *C. albicans* white cells, including the MAPK pathway and the cAMP pathway that mediate filamentation in response to serum, temperature, CO_2_, and starvation [2], [5]. The *C. albicans* MAPK pathway consists of a series of conserved kinases including Cst20, Hst7, and Cek1 that are homologous to *S. cerevisiae* Ste20, Ste7, and Kss1, respectively. The terminal Cek1 kinase activates Cph1, a transcription factor (homologous to *Sc*Ste12) responsible for inducing filamentous growth [6]. The cAMP pathway is similarly activated in response to multiple environmental cues and mediates filamentation via the transcription factor, Efg1 [7]. Together, Cph1 and Efg1 can be regarded as master regulators of *C. albicans* filamentation, and mutants lacking both Cph1 and Efg1 are highly defective in filamentation in white cells [41].

**Figure 5:**
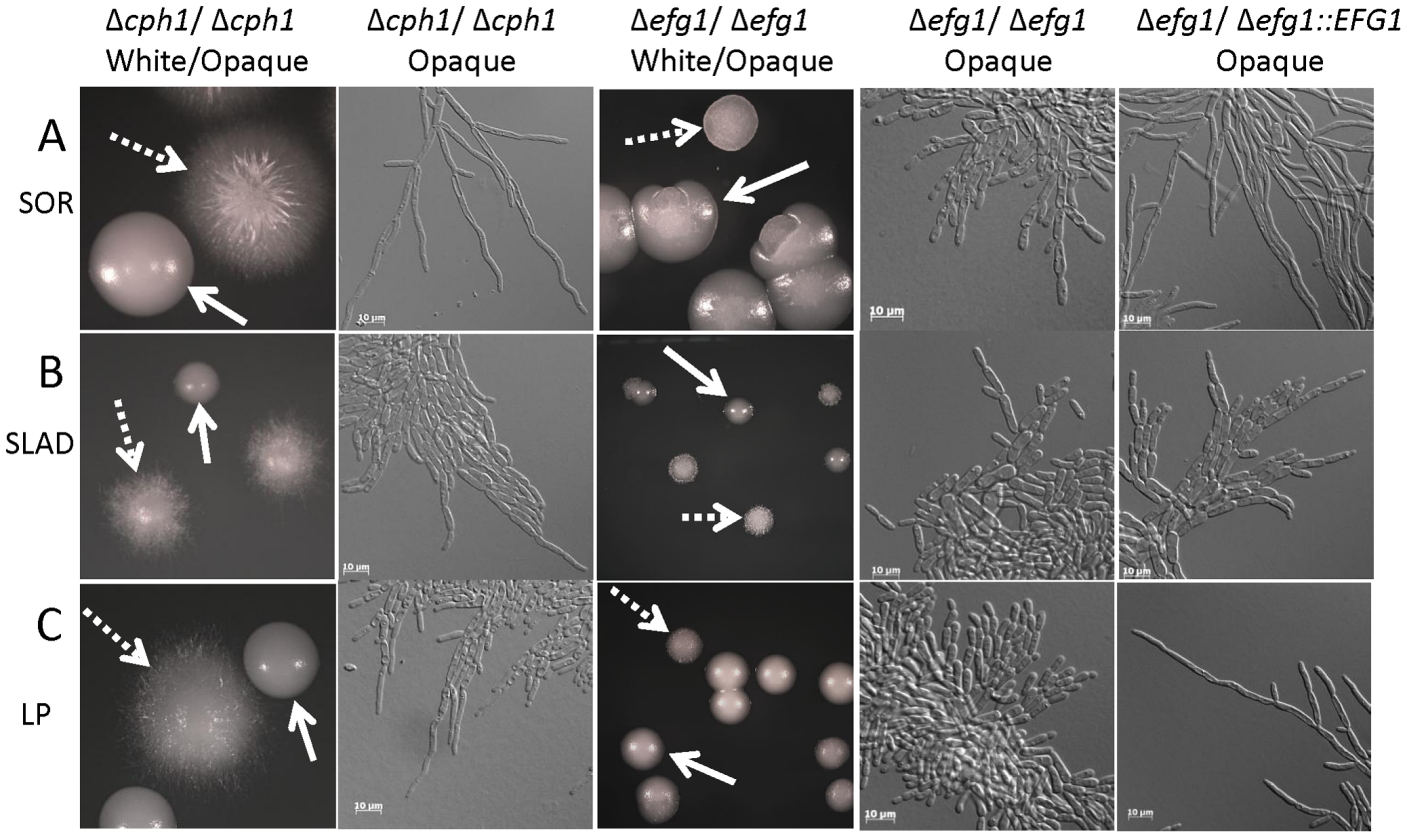
Analysis of the role of the cAMP and MAPK signaling pathways in opaque filamentation. *MTL* **a** strains lacking *EFG1* or *CPH1* transcription factors that mediate the cAMP or MAPK signaling pathway, respectively, were analyzed for their ability to undergo opaque cell filamentation. Loss of *EFG1* resulted in a defect in opaque filamentation under all conditions tested (right panels), while loss of *CPH1* did not have a significant effect on opaque filamentation (left panels). Strains were cultured on SOR (A), SLAD (B) or LP (C) medium for 4 days at 25°C (colonies) or 22 hours at 25°C (cells). *EFG1* mutants were CAY3526 (white) and CAY3292 (opaque), *CPH1* mutants were CAY3524 (white) and CAY3298 (opaque), and the *EFG1* complemented strain was CAY4384 (opaque).

To evaluate the role of the MAPK and cAMP pathways in opaque filamentation, we constructed *MTL* **a**/**a** *Δcph1/Δcph1* and *Δefg1/Δefg1* mutants to analyze MAPK and cAMP signaling, respectively. Construction in an *MTL* homozygous strain was necessary to allow for strains to switch between white and opaque. Loss of Cph1 had little, if any, effect on opaque filamentation under any of the inducing conditions (SOR, MIN, LP, or GlcNAc media), indicating that the MAPK pathway does not play a major role in opaque filamentation. As shown in Figure 5, opaque *Δcph1/Δcph1* colonies appeared similar to wildtype colonies; they exhibited extensive peripheral filamentation and consisted of pseudohyphal or hyphal cells, depending on the inducing medium. We also found that deletion of *CPH1* had only a very modest effect on filamentation phenotypes in white cells (either *MTL* **a**/α or *MTL* **a**/**a** strains, see Figure S4), indicating that the MAPK pathway plays a relatively minor role in regulating filamentation compared to that of the cAMP pathway, consistent with previous observations [41].

The role of *EFG1* in white and opaque cell filamentation was also addressed. We note that loss of *EFG1* has been shown to increase switching from white to opaque [42]–[44], and that switching in *efg1* mutants is regulated by pH [45]. We observed distinct white and opaque *efg1* colonies on SOR, SLAD, and LP media, and loss of Efg1 resulted in decreased opaque cell filamentation under each of these culture conditions. For example, *Δefg1/Δefg1* mutants were unable to form hyphal-like cells when grown on SOR medium and instead grew as chains of opaque cells (Figure 5A). Similarly, whereas wildtype opaque cells formed highly elongated pseudohyphae when grown on SLAD or LP media, *efg1* mutants formed chains of normal looking opaque cells on these media (Figure 5B and C). *Δefg1/Δefg1* mutants also produced smoother colonies compared to filamentous wildtype colonies (Figure 5A–C). Reintegration of the *EFG1* gene into the mutant background restored filamentation, confirming that the mutant phenotype was due to the loss of this gene (Figure 5A–C). Loss of Efg1 was also shown to inhibit filamentation in white *MTL* **a** cells (Figure S4), consistent with its role in white **a**/α cells [7]. These results establish that Efg1 is a master regulator of filamentation in both *C. albicans* white and opaque cells.

### The role of negative transcriptional regulators in white and opaque filamentation

Several negative regulators of filamentation play prominent roles in controlling the yeast-hyphal transition in white cells. These include the transcription factors Nrg1 and Rfg1, as well as the global repressor of gene transcription, Tup1. Loss of any one of these regulators results in white cells growing as hyphae or pseudohyphae under conditions that normally support yeast cell growth [36], [46]–[49]. Somewhat paradoxically, *rfg1* mutants display a defect in hyphae formation under nutrient-limiting conditions [48], while overexpression of *RFG1* promotes pseudohyphal growth [50]. We constructed mutants in each of these factors in switching-competent (*MTL* homozygous) strains to define their role in opaque filamentation.

**Figure 6:**
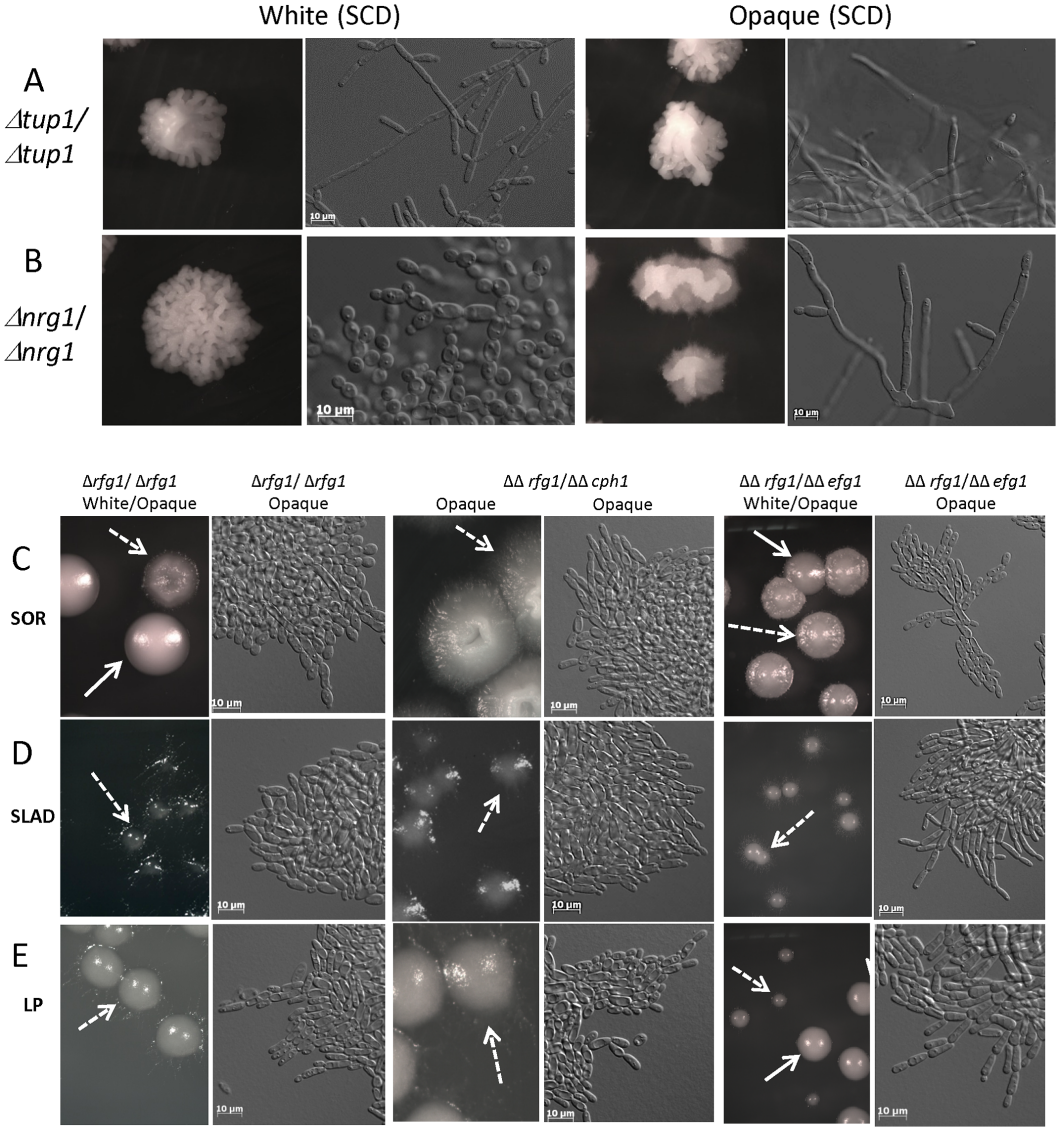
Negative transcriptional regulators of filamentation in white and opaque cells. The roles of the transcription factors Nrg1, Tup1, and Rfg1, which are negative regulators of white cell filamentation, were tested in the opaque program of filamentous growth. Loss of Tup1 (CAY1616, opaque; CAY1550, white) or Nrg1 (CAY1618, opaque; CAY1552, white) resulted in white and opaque cell filamentation even when cells were grown on SCD medium (A and B). In contrast, loss of Rfg1 (CAY3299, opaque; CAY 3528, white) resulted in decreased filamentation on SOR (C), SLAD (D), and LP (E) medium. Loss of both *CPH1* and *RFG1* genes (CAY3151, opaque) resulted in a similar phenotype to *rfg1* mutants (C–E). Loss of both *EFG1* and *RFG1* (CAY2822, opaque; CAY2701 white) generated phenotypes similar to *efg1* and *rfg1* single mutants (C–E). Colonies were grown on the different media for 5 days at 25°C and imaged.

As shown in Figure 6, loss of Nrg1 or Tup1 resulted in filamentation of opaque cells even when cultured on SCD medium, a medium that normally does not induce filamentous growth. Wor1 was constitutively expressed in these cells to drive formation of opaque cells, as *tup1* mutants do not undergo stochastic switching to the opaque state, at least in the WO-1 strain background [51], [52]. Opaque colonies were extremely wrinkled in *nrg1* and *tup1* mutant strains, and consisted mostly of elongated pseudohyphal-like cells with constrictions at sites of cell division (Figure 6A and B). Since the highly filamentous phenotype of the *tup1* mutant is similar between white and opaque cells, we also constructed fluorescence reporters to confirm the phenotypic state of these cells. pOP4-GFP and pWh11-mCherry constructs were employed to indicate the opaque or white state, respectively. As expected, the pOP4-GFP signal was higher in opaque *tup1* cells than in white cells, although basal OP4 expression was evident in white cells (Figure S5B), consistent with previous observations [51]. Conversely, the pWh11-mCherry reporter was expressed in white cells but not opaque cells (Figure S5A). These results establish that opaque *tup1* mutants are maintained in the opaque state and are as filamentous as white *tup1* mutants.

In contrast to Nrg1 and Tup1, loss of Rfg1 reduced filamentation in opaque cells under the conditions tested. For example, growth of *rfg1* mutants on SOR, SLAD or LP media produced very weak filamentation compared to the wildtype strain (Figure 6C–E). In general, *rfg1* mutants grew as chains of opaque cells but cell shape was no longer extremely elongated and was reminiscent of the phenotype of *efg1* mutants (compare Figure 6C–E and Figure 5A–C). We further examined the role of Rfg1 in the context of Cph1 and Efg1 that act in the MAPK and cAMP pathways, respectively. Opaque *Δcph1/Δcph1 Δrfg1/Δrfg1* double mutants exhibited a similar phenotype to *rfg1* mutants, indicating that Cph1 has no effect on opaque filamentation in the *rfg1* background (Figure 6C–E). In addition, opaque *efg1/rfg1* double mutants were analyzed, as both *efg1* and *rfg1* mutants exhibit reduced filamentation. It appeared that *efg1/rfg1* opaque cells resembled *efg1* and *rfg1* single mutants, growing as chains of cells, and that some filamentation was still evident even in the absence of both transcription factors (Figure 6C–E).

Together, these results establish roles for Nrg1, Tup1, and Rfg1 in both white and opaque filamentation programs. Loss of Nrg1 or Tup1 transcriptional repressors results in activation of the program of filamentous growth in white and opaque cells. However, whereas Rfg1 is both a positive and negative regulator of filamentation in white cells depending on the conditions, we observe only a positive role for Rfg1 in promoting opaque cell filamentation.

### Analysis of the Ume6-Hgc1 pathway in opaque filamentation

Recent studies have uncovered Ume6 as a key transcriptional regulator of hyphal and pseudohyphal growth in *C. albicans* white cells. In particular, it was shown that high levels of *UME6* expression drive hyphal formation (and increase virulence), whereas intermediate levels of *UME6* expression resulted in pseudohyphal growth [29]. The cyclin-related protein Hgc1 is an important downstream target of Ume6 and mediates agar invasion, hyphal extension, and formation of true septa [53]. Hgc1 functions acts as part of a Hgc1/Cdc28 complex that promotes filamentation by phosphorylating Rga2, a Cdc42 GAP protein, which in turn activates Cdc42 and drives actin polymerization [54]. In addition, both *UME6* and *HGC1* are negatively regulated by the Nrg1 and Tup1 transcription factors discussed above [25], [55]. Mutants in *UME6* and *HGC1* were constructed and tested in *MTL* **a**/**a** strain backgrounds to determine their contribution to opaque filamentation phenotypes.

**Figure 7:**
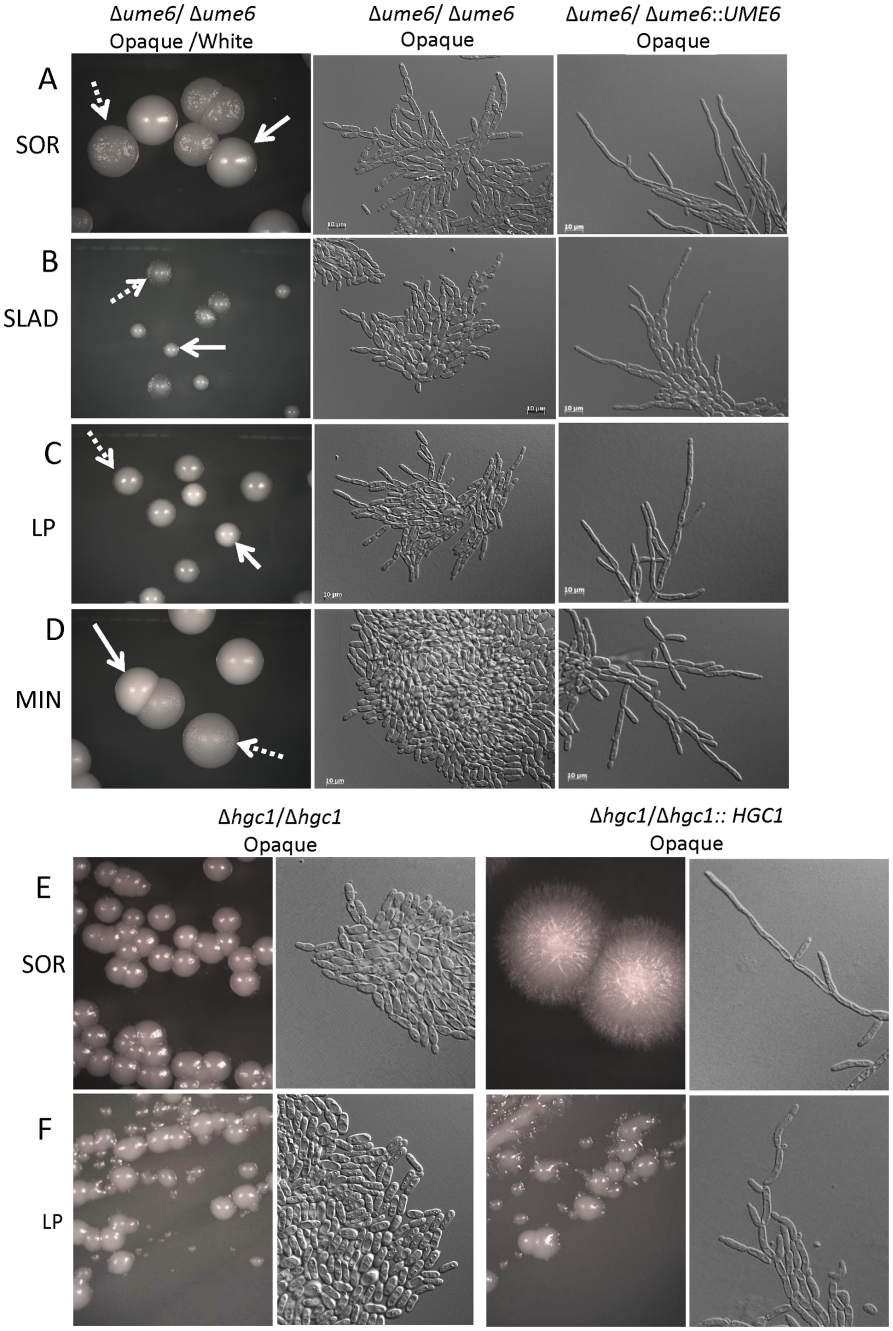
Role of the Ume6-Hgc1 pathway in opaque cell filamentation. The transcription factor Ume6 and the G1 cyclin-related protein Hgc1 were analyzed for their potential role(s) in filamentation in opaque cells. Mutant or complemented opaque strains were incubated on SOR (A and E), SLAD (B), LP (C and F), or MIN (D) medium for 4 days at 25°C and colony morphologies determined. Cell morphologies were photographed after 25 hours incubation. Loss of Ume6 (CAY1571) or Hgc1 (CAY4193) compromised opaque filamentation under each of the tested conditions, and filamentation was restored in the complemented strains CAY3697 (*UME6* addback) and CAY4197 (*HGC1* addback), respectively. Strain DK340 was the white *ume6* mutant used.

Deletion of *UME6* resulted in a marked decrease in opaque cell filamentation under each of the tested media conditions. Thus, growth of opaque *ume6* mutants on SOR, LP, or MIN media generated chains of cells but cells were no longer highly elongated and no hyphal-like cells were observed. In fact, opaque *ume6* cells resembled those of *efg1* and *rfg1* mutants (compare Figure 7 and Figure 6C–D). These results show strong parallels between white and opaque cells, as deletion of *UME6* also compromises white cell filamentation [25], [55].

In white cells, *UME6* overexpression also drives cells into the hyphal form [29]. We therefore addressed whether *UME6* overexpression is sufficient to induce opaque cell filamentation by using a strain in which *UME6* is under the control of the *Escherichia coli tet* operator (*tetO*). The constructed strain also expresses an *E. coli tet* repressor-*Saccharomyces cerevisiae* Hap4 activation domain fusion protein. As a result, the *UME6* gene is turned off in the presence of doxycycline, but is highly induced in the absence of doxycycline [29]. We found that overexpression of *UME6* induced filamentous growth in both white and opaque cells, establishing *UME6* as a master regulator of filamentation in both phenotypic states (Figure S6).

The role of *HGC1* in opaque cell filamentation was also examined. These mutants were constructed in the P37005 background of *C. albicans* that is a natural *MTL* **a**/**a** isolate. The wildtype P37005 opaque cells underwent filamentation in response to inducing conditions, including on SOR and LP media. In contrast, P37005 *Δhgc1/Δhgc1* mutants showed a marked defect in opaque filamentation, as these strains were unable to form filaments when grown on SOR or LP media (Figure 7E and F). The defect in *hgc1* mutant filamentation was restored by complementation with the wildtype *HGC1* gene (Figure 7E and F). Together, these results indicate a shared role for *UME6* and *HGC1* in promoting filamentation in both white and opaque cells.

### Analysis of additional white cell filamentation regulators for roles in opaque cell filamentation

The Ras1 protein plays a prominent role in white cell filamentation and acts upstream of both the MAPK and cAMP pathways. Mutants in *ras1* show a severe defect in hyphal growth in white cells under multiple conditions, while strains expressing a dominant active Ras1 mutation (Ras1^G13V^) show enhanced hyphal formation [56]. Here, we tested the phenotype of *Δras1/Δras1* mutants in opaque cells and observed a defect in opaque filamentation on SOR and LP media (Figure S7), consistent with cAMP signaling being necessary for filamentous growth (compare to the *efg1* mutant phenotype, Figure 5). Curiously, expression of the constitutively active Ras1^G13V^ allele also partially suppressed filamentation on LP and SOR media (Figure S7). Thus, either loss of *RAS1* function or hyperactive Ras1 activity appears to decrease filamentation of opaque cells.

Other pathways that regulate white cell filamentation include the Cph2/Tec1 pathway. Both of these factors are transcription factors and Cph2 is necessary for Tec1 expression, which in turn upregulates genes involved in hyphal development [57], [58]. Mutants in *cph2* and *tec1* were examined in the opaque phase but these mutants had no significant effect on filamentous growth under any of the tested conditions (Figure S8).

White cells are also induced to undergo filamentation when embedded in soft agar, and this program is dependent on the Czf1 transcription factor [59]. Opaque cells similarly underwent increased filamentation when cultured under embedded conditions (data not shown). Embedded conditions therefore represent an environmental cue that is conducive to inducing filamentation in both white and opaque cells. Interestingly, embedded growth is also one of the few filament-inducing conditions that is effective at 25°C for white cells [59]. As Czf1 promotes white filamentation under embedded conditions, opaque *czf1* mutants were analyzed. This required overexpression of *WOR1* to generate opaque cells, as *czf1* mutants exhibit very low rates of white-to-opaque switching [44]. Opaque *czf1* mutants produced an unusual ‘hyper-branching’ phenotype when grown on the surface on several media, including SLAD, LP and SOR medium (Figure S9). *czf1* mutant opaque cells grew as highly branched chains that were clearly distinguishable from all other filamentation phenotypes. Czf1 is a therefore a regulator of filamentation in opaque cells, even when grown under non-embedded conditions.

### Transcriptional profiling of filamentous opaque cells

Gene expression of filamentous white cells has been defined during growth in serum medium at 37°C [36], [60]. We set out to similarly define the transcriptional profile(s) of filamentous opaque cells, and to compare these profiles to those of filamentous white cells. Two medium conditions were used to induce opaque cell filamentation: low phosphate (LP) medium at 12 hours and sorbitol (SOR) medium at 16 hours (both at 25°C), as robust filamentation was observed at these time points. Expression was compared to that in SCD medium (non-filament inducing). We also compared gene expression profiles between white and opaque cells under these culture conditions.

First of all, we note that the master transcriptional regulators of the phenotypic switch were differentially expressed between white and opaque phenotypes under these culture conditions. Thus, *WOR1*, *WOR2*, and *CZF1* were expressed at higher levels in opaque cells, while *EFG1* was expressed at a higher level in white cells (data not shown). These results establish that cells are exhibiting the classical opaque transcriptional pattern when cultured in LP or SOR medium.

Comparison of filamenting and non-filamenting opaque cells revealed that 1188 genes were differentially expressed (by SAM) between LP and SCD medium, while 341 genes were differentially regulated between SOR and SCD medium. More specifically, 445 genes were induced and 743 genes repressed (>3-fold) in LP medium, while 143 genes were induced and 198 genes repressed in SOR medium. Presumably, these expression changes include many genes that are regulated by the nutritional change, as well as genes that directly mediate the transition from yeast to filamentous growth.

**Figure 8:**
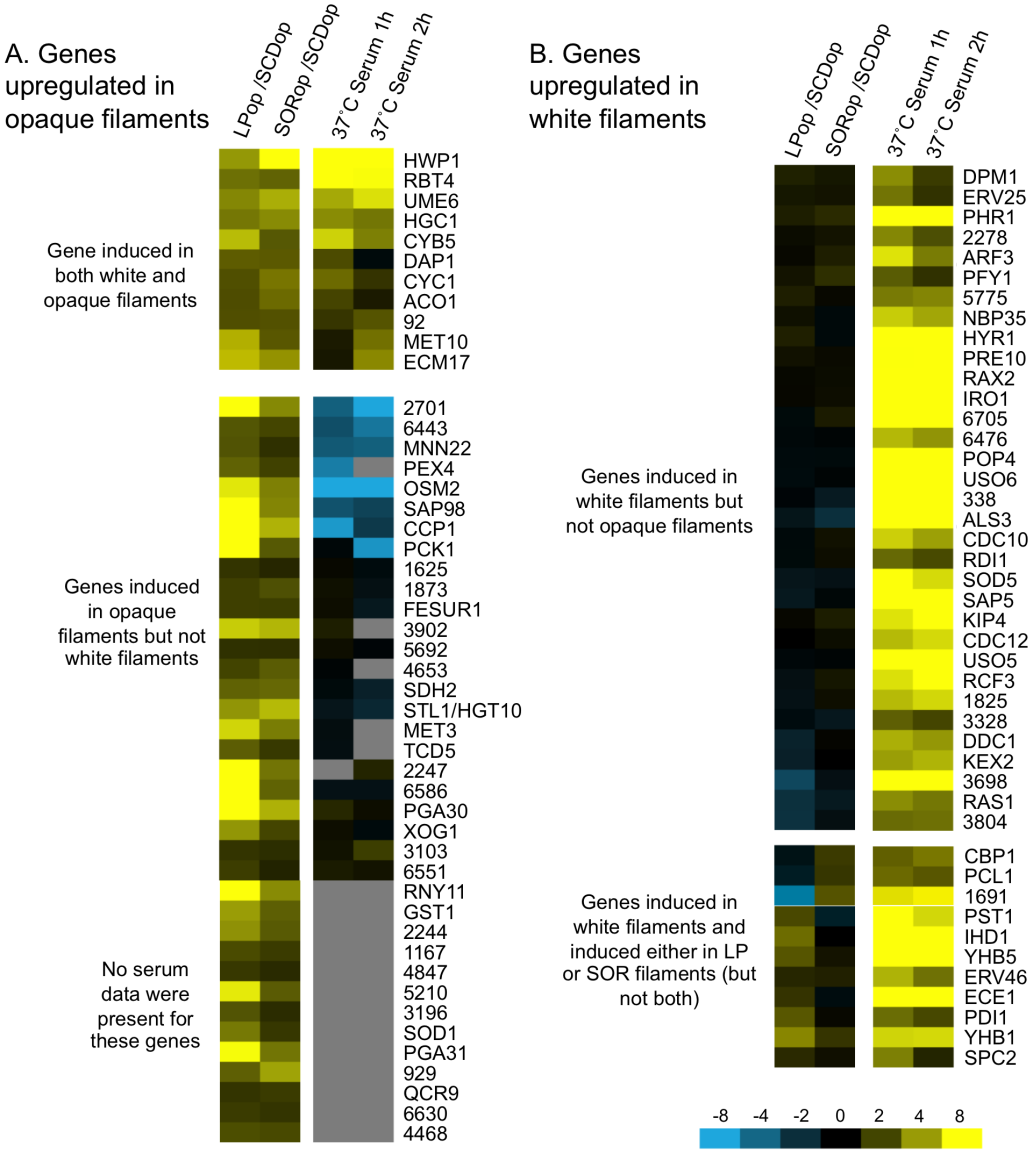
Transcriptional profiling of opaque filamentation. *MTL* **a** white (RBY717) or opaque (CAY2903) strains were incubated in SCD medium, a non-filament inducing condition, or sorbitol (SOR) and low phosphate (LP) media, conditions that specifically induce filamentation of opaque cells but not white cells. Cells were harvested after 12 hours growth (LP medium) or 16 hours growth (SOR medium) and cDNA prepared and hybridized against custom *C. albicans* ORF microarrays. (A) Comparative expression of opaque cell filamentation genes and white hyphal genes. Columns 1 and 2 represent opaque filamentation genes in LP and SOR media (relative to SCD controls) that passed SAM filters. Columns 3 and 4 represent hyphal-induced genes in white cells (serum-treated cells at 1 or 2 h compared to untreated cells) using data from Kadosh and Johnson [36]. Top panel; genes induced in both opaque and white filaments. Bottom panel, genes induced in opaque filaments but not white filaments. Grey boxes indicate no data available. (B) Genes expressed in white hyphal cells but not expressed in filamenting opaque cells. Top panel; genes induced in white hyphal cells only. Bottom panel, genes induced in white hyphal cells and either in opaque LP filaments or SOR filaments (but not both). op, opaque cells; wh, white cells. Gene numbers refer to orf19 gene identifiers (e.g. 1774 refers to orf19.1774).

Comparison of gene expression profiles revealed that a core set of 48 genes was induced during filamentous growth of opaque cells in both SOR and LP medium (see Table S2). These genes were compared to those induced during white cell filamentation in serum at 37°C (Figure 8). In general, overlap between white and opaque filamentation profiles was limited, with most genes specific to one program or the other. Thus, many of the genes characteristic of hyphal formation in white cells, including *ALS3*, *HYR1*, *PHR1*, and *SAP5*, were not induced in filamentous opaque cells (Figure 8B). In fact, of the 55 genes induced in white cell hyphae, only 11 were induced in filamentous opaque cells in LP and SOR media. Significantly, one gene that was highly induced during both white and opaque filamentation was the key transcriptional regulator *UME6*. This result is consistent with the genetic requirement for this factor for filamentous growth in both white and opaque cells.

In general, most hyphal-specific genes in white cells were not induced in filamenting opaque cells, and conversely opaque cells expressed filamentation genes not induced in white cells. In fact, several genes induced during opaque filamentation were repressed in white hyphal cells, including *MNN22*, *OSM2*, *PCK1*, and *SAP98* (Figure 8B), while data for a number of opaque filamentation genes was not present in the white expression data set. Several opaque-specific filamentation genes are of interest, including *PGA30* and *PGA31* that encode putative GPI-anchored cell wall proteins. Upregulation of these genes suggests that filamentous opaque cells could exhibit altered adherence compared to yeast cells. A similar phenomenon has been observed in white cells, where expression of hyphal-specific surface proteins (e.g. *ALS3*, *ALS10*, *ECE1*, and *HWP1*) mediates increased adhesion of hyphae to host cells and promotes biofilm formation [61], [62]. We note that *HWP1* expression was increased in both white and opaque filamentous cells (Figure 8B) and was also induced in opaque cells forming polarized mating projections [16], consistent with the model that *HWP1* expression is directly regulated by actin dynamics during the morphological transition from yeast to polarized growth [63].

Together, these analyses reveal that the gene expression profiles of filamentous white and opaque cells are distinct, with only a limited number of factors induced in both programs of filamentation. However, the induction of *UME6* in both gene profiles is consistent with a key role for this transcription factor in regulating filamentation in both white and opaque cell types.

## Discussion

*C. albicans* is multimorphic – it grows in a variety of morphological forms and the ability to switch between these forms underlies its ability to colonize and infect diverse niches in the mammalian host. Here, we address the relationship between two distinct forms of phenotypic plasticity; white-opaque switching and the transition between yeast and filamentous forms. We report that white and opaque cells can both undergo filamentation but do so in response to distinct environmental cues. Furthermore, we define the transcriptional profile of filamentous opaque cells and reveal marked differences with genes regulated by the yeast-hyphal transition in white cells. We also compare and contrast the signal transduction pathways that regulate filamentation in *C. albicans* white and opaque cell types.

### Differential regulation of filamentous growth in *C. albicans* white and opaque cells

Our results demonstrate that the white-opaque switch plays a key role in regulating the yeast-hyphal transition in *C. albicans*. Thus, two of the best-studied programs regulating morphogenesis in *C. albicans* are interconnected, indicating overlap of the regulatory mechanisms involved in these programs. We show that opaque cells can form filamentous cells and do so in response to different environmental cues than those that induce filamentous growth in white cells. Thus, whereas serum, neutral pH, nutrient deprivation and high temperature are signals that induce filamentation in white cells, these stimuli do not induce filamentation in opaque cells (Figure 1). In contrast, opaque cells undergo filamentation in response to distinct cues, including sorbitol or low phosphate medium that do not induce filamentous growth in white cells (Figure 2). These results establish that white and opaque states are differentially programmed with respect to the integration of environmental signals for filamentous growth.

### Genetic regulation of opaque filamentation

The regulation of filamentous growth in *C. albicans* white cells has been the subject of extensive studies (reviewed in [2], [5]). We examined whether the regulatory pathways controlling white cell filamentation, including MAPK and cAMP pathways, also function to regulate opaque cell filamentation. We show that Efg1, the terminal transcription factor in the cAMP pathway, and Ume6, a transcription factor that acts downstream of Efg1 [29], [53], [55], are key regulators of filamentation in opaque cells. Furthermore, overexpression of *UME6* was sufficient to induce opaque cell filamentation, as it is in white cells [29]. We also observed roles for the positive regulator Hgc1 and the negative regulators Nrg1-Tup1 in opaque cell filamentation, similar to their established roles in white cell filamentation. Filamentous growth in white cells can be either positively or negatively regulated by Rfg1 depending on the conditions [48], and we found that *rfg1* mutants were defective for filamentation in opaque cells. Thus, this transcription factor also plays a role in regulating filamentation in both white and opaque cells.

**Figure 9:**
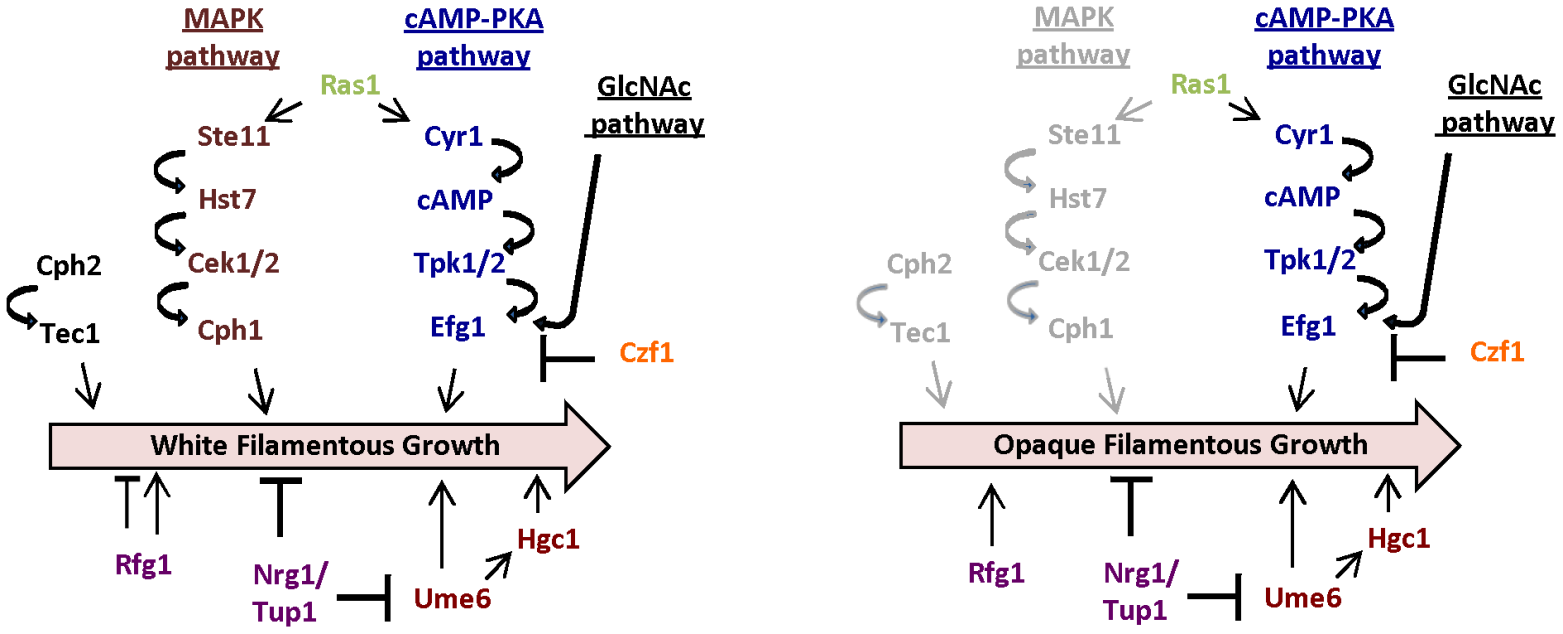
Regulation of filamentous growth in *C. albicans* white and opaque cells. The central programs regulating filamentation in white cells are shown, including the two core pathways of MAPK and cAMP-PKA signaling. White cell filamentation is also negatively regulated by the transcription factors Tup1 and Nrg1, and positively regulated by Ume6 in concert with the cyclin-like protein Hgc1. Filamentation in opaque cells is regulated by many of the same signaling pathways. In particular, Efg1 and Ume6 appear to be master regulators of filamentous growth in both white and opaque cells. However, there was no detectable role for MAPK signaling through Cph1, or for signaling via Cph2/Tec1, under the conditions used for inducing opaque filamentation.

We also examined several other regulators of white cell filamentation for potential roles in opaque cell filamentation. Cph1, the master regulator of the MAPK pathway, did not influence opaque cell filamentation and we also observed that this factor has only a subtle role in white cell filamentation in the wildtype strain background. Similarly, Cph2 and Tec1 did not affect opaque filamentation, while deletion of Czf1 resulted in a hyper-branching phenotype specifically in opaque cells at 25°C. Taken together, our findings indicate that white and opaque filamentation occurs in response to different environmental stimuli, and generates different transcriptional responses (discussed below), but that genetic regulation of these programs involves many of the same signaling pathways in both phenotypic states (see model in Figure 9).

### Contrasting temperature regulation of filamentation in white and opaque cells

Filamentous growth in white cells is often dependent upon elevated temperatures, with several hyphal-inducing conditions requiring a temperature of 37°C for efficient filamentation [2], [5]. We found that filamentous growth in opaque cells exhibited the opposite dependence on temperature; opaque cells underwent filamentation efficiently at 25°C while filamentation was reduced at 30°C or 37°C. This was not due to opaque cells switching back to the white form at 37°C, as opaque cells were stably maintained by constitutive expression of *WOR1*, the master regulator of the opaque state [33]–[35]. In white cells, the molecular chaperone Hsp90 has recently been implicated in thermal regulation of the yeast-hyphal transition, as raised temperatures compromised Hsp90-mediated repression of hyphal formation [8]. In contrast to white cells, we found that inhibition of Hsp90 failed to elicit a large-scale change in morphology, although this could be due, at least in part, to the increased sensitivity of opaque cells to geldanamycin. These results reveal that elevated temperatures and/or decreased Hsp90 activity typically promote hyphal growth in white cells, while lower temperatures promote filamentation in opaque cells. Our findings have direct implications for pathogenicity by white/opaque cells, as they are likely to filament in different niches in response to different environmental cues, as discussed below.

### Polarized growth and opaque filamentation

While this study revealed a novel program of filamentous growth in opaque cells, it is noted that polarized growth in opaque cells is not a new phenomenon. Opaque cells are the mating-competent form of *C. albicans* and respond to pheromones by forming mating projections that can be many times the length of the original opaque cell [16], [17]. Furthermore, opaque mating projections contain a Spitzenkörper-like structure in which the myosin regulatory light chain protein, Mlc1, localizes to a characteristic ball at the growing tip of the cell [18], [64]. The fact that opaque cells can form a Spitzenkörper indicates that these cell types undergo highly polarized growth by a mechanism similar to that in truly filamentous fungi. The discovery of environmental conditions that induces opaque filamentation now opens the door for further exploration of the regulation of opaque filamentation.

### Comparison of white and opaque gene expression during filamentation

Gene expression profiles of filamenting white and opaque cells were distinct, with only 11 genes induced in both programs, while a further 44 genes were induced specifically in white hyphal cells and 37 genes induced only in opaque filamentous cells (Figure 8). One gene that was highly induced in both white and opaque states was the transcription factor *UME6*. As discussed above, *UME6* is a critical regulator of filamentous growth in opaque cells, similar to its established role in white cells, and expression profiling confirms that this gene is co-regulated by morphogenesis in both cell types.

Interestingly, comparison of white-opaque regulated genes reveals that several regulators of filamentation are differentially expressed between white and opaque cells. For example, *UME6*, *HGC1*, *NRG1*, and *EFG1*, are white-opaque regulated genes; the first two genes are expressed at significantly higher levels in opaque cells, while the latter two genes show elevated expression in white cells (see Figure S10). *EFG1* is itself a master regulator both of the white-opaque phenotypic switch and of filamentous growth [3], [43], [44]. It is therefore apparent that the transcriptional regulation of white/opaque phenotypes and that of filamentous growth are highly integrated, as previously suggested [65]. Presumably, the differential expression of these key regulators is at least partially responsible for the different propensities of white and opaque cells to filament in response to different environmental cues.

### The role of white and opaque filamentation during infection

The yeast-hyphal transition is critical for infection by *C. albicans* white cells, where hyphae are more adherent and invasive than yeast-form cells. Despite this, questions remain as to the exact role of the hyphal structure during infection, and whether it is the genes that are co-regulated with the morphological switch that are critical for virulence or the hyphal structure itself [2]. In contrast, little is known about the potential for opaque cell filaments to promote pathogenesis. Early studies indicated that opaque cells are more effective at causing skin infections than white cells, and that opaque filamentation was induced on human skin epithelium [11], [12], [15]. Mating between opaque cells has also been shown to occur in this niche, indicating that the skin may represent a natural site for opaque colonization [66].

It is also possible that opaque filamentation occurs in other environmental niches, including those not associated with colonization and infection of the mammalian host. Future studies will examine both *in vitro* and *in vivo* conditions to determine those capable of inducing the program of filamentation in opaque cells. It is therefore an open question as to the role of the filamentous program in *C. albicans* opaque cells in infection and disease, and whether this program parallels that in white cells in promoting tissue destruction and pathogenesis.

## Supporting Information

Figure S1 **Natural opaque cells undergo filamentation in response to environmental cues.** Culture conditions induce filamentous growth in wildtype opaque cells as well as in opaque-locked cells (see Figure 2). Strain RBY731 (opaque form of strain RBY717) was grown on (A) SCD (B) low phosphate (LP), (C) low nitrogen (SLAD), or (D) sorbitol (SOR) medium at 25°C. These culture conditions do not induce filamentation in white cells. Panels show colony morphologies from mixed white/opaque populations (solid arrow, white colonies; dashed arrow, opaque colonies) after 4 days growth. Additional panels show DIC images of white and opaque cells, as well as calcofluor white (CW)-stained cells after 22 hours growth. Scale bar, 10 µm.
(TIFF)

Figure S2 **Opaque cells undergo filamentation in liquid culture media.** Opaque cells (strain CAY2903) undergo filamentation when cultured in liquid media, in addition to growth on solid media (Figure 2). Images were taken after 16 hours incubation at 25°C. (A), SCD, (B), SOR, (C), LP, (D), MIN, and (E) SLAD medium. Opaque cell filamentation is strongest in liquid SOR and LP media.
(TIF)

Figure S3 **Contrasting Hsp90-mediated regulation of morphogenesis in white and opaque cells.** White cells (RBY717) treated with the Hsp90 inhibitor geldanamycin (GdA) were induced to undergo filamentous growth at 30°C, but not 25°C. In contrast, opaque cells (CAY2903) did not undergo efficient filamentation when incubated with GdA at either temperature. Cells were grown at 25°C (A and C) or 30°C (B and D) and treated with 10 µM GdA for 12 hours.
(TIFF)

Figure S4 **Comparison of** ***cph1*** **and** ***efg1*** **mutant phenotypes in white** ***MTL*** **a/α and** ***MTL*** **a strains.** All strains were grown on Spider medium at 30°C for 4 days and photographed. (A) Wildtype white **a**/α, (B) wildtype white **a**/**a** strain (RBY717), (C) *cph1* white **a**/α strain (CJN2741), (D) *cph1* white **a**/Δα strain (CAY4479), (E) *efg1* white **a**/α strain (CAY4522), (F) *efg1* white **a**/Δα strain (CAY3526). In both *MTL* heterozygous and *MTL* homozygous strains the *cph1* mutant had a subtle defect in filamentation while the *efg1* mutant had a marked defect in filamentation.
(TIF)

Figure S5 **Deletion of** ***TUP1*** **induces filamentous growth in both white and opaque cells.** The role of the transcription factor Tup1 was tested in both white and opaque programs of filamentous growth using fluorescent reporters to confirm the phenotypic state of the cell. (A) White *tup1* mutants expressing a white-specific reporter (p*WH11*-mCherry) or an opaque-specific reporter (p*OP4*-GFP) in strains CAY4356 or CAY4353, respectively. Strong expression of the *WH11* gene (high mCherry levels) confirms that white *tup1* mutants are undergoing filamentation. (B) Opaque *tup1* mutants expressing white and opaque reporter constructs in strains CAY4492 and CAY4291. Strong expression of the opaque-specific *OP4* reporter confirms that *tup1* mutants are propagating in the opaque state and undergoing constitutive filamentous growth similar to that of white cells. Cells were grown for 16 h in SCD medium and photographed. Scale bar, 10 µm.
(TIF)

Figure S6 **Induction of** ***UME6*** **expression induces filamentation in both white and opaque cells.** The *UME6* gene was placed under the control of the *tetO* operator in a strain expressing the *E. coli tet* repressor - *S. cerevisiae* Hap4 activation domain fusion protein. In the presence of Dox (doxycycline) the *UME6* gene is repressed (A and C), while in the absence of Dox the *UME6* gene is induced (B and D). In both white (CAY4504) and opaque (CAY4502) cells filamentous growth occurred when grown on YPD without doxycycline. Colonies were grown for 6 days at 25°C. Scale bar, 10 µm.
(TIF)

Figure S7 **Deletion or overexpression of** ***RAS1*** **leads to a defect in opaque filamentation.** Wildtype cells (expressing a Ras1-GFP fusion protein), *Δras1/Δras1* mutants, or cells expressing a constitutively active Ras1 allele (G13V) were compared for their ability to undergo filamentation in the opaque state. Both *ras1* mutants and strains expressing hyperactive *RAS1* alleles showed decreased filamentation on LP and SOR medium relative to the control strain. Strains were incubated on media for 4 days at 25°C. Strains used were wildtype white cells (CAY3749), opaque cells (CAY3619), *ras1* white cells (CAY2723), *ras1* opaque cells (CAY2795), and constitutively active Ras1 white cells (CAY3751) and opaque cells (CAY3621).
(TIF)

Figure S8 **Analysis of the role of Cph2 and Tec1 in opaque filamentation.** Mutants lacking (A) Tec1 and (B) Cph2 were analyzed for opaque filamentation phenotypes. Neither of these factors appeared to play a significant role in filamentous growth in opaque cells. Strains were incubated on media for 22 hours (cell images) or 4 days (colony images) at 25°C. Cph2 mutants used were CAY2091 (white cells) and CAY3296 (opaque cells). Tec1 mutants used were CAY2646 (white cells) and CAY2688 (opaque cells). Solid arrow, white cells; dashed arrow, opaque cells. Scale bar, 10 µm.
(PDF)

Figure S9 **Analysis of the role of Czf1 in opaque filamentation.** Opaque cells (CAY3294) lacking the transcription factor Czf1 were found to exhibit a hyper-branching phenotype when grown on LP, SLAD and SOR medium. In contrast, white *czf1* mutants (CAY3522) did not exhibit this phenotype. Solid arrow, white colonies; dashed arrow, opaque colonies. Cells were imaged after 22 h and colonies after 4 d at 25°C.
(TIF)

Figure S10 **Comparative expression of yeast-hyphal regulators between white and opaque cells.** Gene expression was compared for multiple yeast-hyphal regulators between white and opaque cells and revealed that many of these regulators are expressed in a phase-specific pattern. For example, the regulators *CZF1*, *UME6*, *HGC1*, *CEK1*, and *CEK2* are expressed at an elevated level (>4-fold) in opaque cells compared to white cells. In contrast, other established yeast-hyphal regulators including *EFG1* and *NRG1* are expressed more in white cells than opaque cells (>3-fold). Finally some regulators are not differentially expressed between white and opaque cells (e.g. *CPH1* and *RFG1*). Fold change in expression is shown in parentheses. Data adapted from RNA-seq analysis in Tuch *et al.* [30].
(TIF)

Table S1 **Oligonucleotides used in this study.**
(DOCX)

Table S2 **Genes induced during filamentous growth in opaque cells.** Table shows genes induced during filamentous growth of opaque cells in both LP and SOR media.
(XLSX)

Text S1 **SAM_for LP op v. SCD op.** File includes SAM analysis of genes differentially expressed in LP medium compared to SCD medium for opaque cells.
(XLSX)

Text S2 **SAM_for SOR op v. SCD op.** File includes SAM analysis of genes differentially expressed in SOR medium compared to SCD medium for opaque cells.
(XLSX)

## References

[1] Sudbery P, Gow N, Berman J (2004) The distinct morphogenic states of *Candida albicans* . Trends Microbiol 12: 317–324. 15223059 10.1016/j.tim.2004.05.008

[2] Sudbery PE (2011) Growth of *Candida albicans* hyphae. Nat Rev Microbiol 9: 737–748. 21844880 10.1038/nrmicro2636

[3] Lo HJ, Kohler JR, DiDomenico B, Loebenberg D, Cacciapuoti A, et al (1997) Nonfilamentous *C. albicans* mutants are avirulent. Cell 90: 939–949. 9298905 10.1016/s0092-8674(00)80358-x

[4] Lorenz MC, Bender JA, Fink GR (2004) Transcriptional response of *Candida albicans* upon internalization by macrophages. Eukaryot Cell 3: 1076–1087. 15470236 10.1128/EC.3.5.1076-1087.2004 PMC522606

[5] Shapiro RS, Robbins N, Cowen LE (2012) Regulatory circuitry governing fungal development, drug resistance, and disease. Microbiol Mol Biol Rev 75: 213–267. 10.1128/MMBR.00045-10 PMC3122626 21646428

[6] Liu H, Kohler J, Fink GR (1994) Suppression of hyphal formation in *Candida albicans* by mutation of a *STE12* homolog. Science 266: 1723–1726. 7992058 10.1126/science.7992058

[7] Stoldt VR, Sonneborn A, Leuker CE, Ernst JF (1997) Efg1p, an essential regulator of morphogenesis of the human pathogen *Candida albicans*, is a member of a conserved class of bHLH proteins regulating morphogenetic processes in fungi. EMBO J 16: 1982–1991. 9155024 10.1093/emboj/16.8.1982 PMC1169801

[8] Shapiro RS, Uppuluri P, Zaas AK, Collins C, Senn H, et al (2009) Hsp90 orchestrates temperature-dependent *Candida albicans* morphogenesis via Ras1-PKA signaling. Curr Biol 19: 621–629. 19327993 10.1016/j.cub.2009.03.017 PMC2735497

[9] Slutsky B, Staebell M, Anderson J, Risen L, Pfaller M, et al (1987) “White-opaque transition”: a second high-frequency switching system in *Candida albicans* . J Bacteriol 169: 189–197. 3539914 10.1128/jb.169.1.189-197.1987 PMC211752

[10] Miller MG, Johnson AD (2002) White-opaque switching in *Candida albicans* is controlled by mating-type locus homeodomain proteins and allows efficient mating. Cell 110: 293–302. 12176317 10.1016/s0092-8674(02)00837-1

[11] Kvaal C, Lachke SA, Srikantha T, Daniels K, McCoy J, et al (1999) Misexpression of the opaque-phase-specific gene *PEP1* (*SAP1*) in the white phase of *Candida albicans* confers increased virulence in a mouse model of cutaneous infection. Infect Immun 67: 6652–6662. 10569787 10.1128/iai.67.12.6652-6662.1999 PMC97079

[12] Kvaal CA, Srikantha T, Soll DR (1997) Misexpression of the white-phase-specific gene *WH11* in the opaque phase of *Candida albicans* affects switching and virulence. Infect Immun 65: 4468–4475. 9353021 10.1128/iai.65.11.4468-4475.1997 PMC175642

[13] Lohse MB, Johnson AD (2008) Differential phagocytosis of white versus opaque *Candida albicans* by Drosophila and mouse phagocytes. PLoS One 3: e1473. 18213381 10.1371/journal.pone.0001473 PMC2198939

[14] Ernst JF (2000) Transcription factors in *Candida albicans* - environmental control of morphogenesis. Microbiology 146 Pt 8: 1763–1774. 10931884 10.1099/00221287-146-8-1763

[15] Anderson J, Cundiff L, Schnars B, Gao MX, Mackenzie I, et al (1989) Hypha formation in the white-opaque transition of *Candida albicans* . Infect Immun 57: 458–467. 2643570 10.1128/iai.57.2.458-467.1989 PMC313119

[16] Bennett RJ, Uhl MA, Miller MG, Johnson AD (2003) Identification and characterization of a *Candida albicans* mating pheromone. Mol Cell Biol 23: 8189–8201. 14585977 10.1128/MCB.23.22.8189-8201.2003 PMC262406

[17] Lockhart SR, Daniels KJ, Zhao R, Wessels D, Soll DR (2003) Cell Biology of Mating in *Candida albicans* . Eukaryot Cell 2003: 49–61. 10.1128/EC.2.1.49-61.2003 PMC141171 12582122

[18] Chapa YLB, Lee S, Regan H, Sudbery P (2011) The mating projections of *Saccharomyces cerevisiae* and *Candida albicans* show key characteristics of hyphal growth. Fungal Biol 115: 547–556. 21640318 10.1016/j.funbio.2011.02.001

[19] Guthrie C, Fink GR (1991) Guide to Yeast Genetics and Molecular Biology. San Diego: Academic Press.

[20] Reuss O, Vik A, Kolter R, Morschhauser J (2004) The *SAT1* flipper, an optimized tool for gene disruption in *Candida albicans* . Gene 341: 119–127. 15474295 10.1016/j.gene.2004.06.021

[21] Gimeno CJ, Fink GR (1992) The logic of cell division in the life cycle of yeast. Science 257: 626. 1496375 10.1126/science.1496375

[22] Bedell GW, Soll DR (1979) Effects of low concentrations of zinc on the growth and dimorphism of *Candida albicans*: evidence for zinc-resistant and -sensitive pathways for mycelium formation. Infect Immun 26: 348–354. 387610 10.1128/iai.26.1.348-354.1979 PMC414618

[23] Basso LR Jr, Bartiss A, Mao Y, Gast CE, Coelho PS, et al (2010) Transformation of *Candida albicans* with a synthetic hygromycin B resistance gene. Yeast 27: 1039–1048. 20737428 10.1002/yea.1813 PMC4243612

[24] Noble SM, Johnson AD (2005) Strains and strategies for large-scale gene deletion studies of the diploid human fungal pathogen *Candida albicans* . Eukaryot Cell 4: 298–309. 15701792 10.1128/EC.4.2.298-309.2005 PMC549318

[25] Banerjee M, Thompson DS, Lazzell A, Carlisle PL, Pierce C, et al (2008) *UME6*, a novel filament-specific regulator of *Candida albicans* hyphal extension and virulence. Mol Biol Cell 19: 1354–1365. 18216277 10.1091/mbc.E07-11-1110 PMC2291399

[26] Homann OR, Dea J, Noble SM, Johnson AD (2009) A phenotypic profile of the *Candida albicans* regulatory network. PLoS Genet 5: e1000783. 20041210 10.1371/journal.pgen.1000783 PMC2790342

[27] Sherwood RK, Bennett RJ (2008) Microtubule motor protein Kar3 is required for normal mitotic division and morphogenesis in *Candida albicans* . Eukaryot Cell 7: 1460–1474. 18586948 10.1128/EC.00138-08 PMC2547067

[28] Hernday AD, Noble SM, Mitrovich QM, Johnson AD (2012) Genetics and molecular biology in *Candida albicans* . Methods Enzymol 470: 737–758. 10.1016/S0076-6879(10)70031-8 20946834

[29] Carlisle PL, Banerjee M, Lazzell A, Monteagudo C, Lopez-Ribot JL, et al (2009) Expression levels of a filament-specific transcriptional regulator are sufficient to determine *Candida albicans* morphology and virulence. Proc Natl Acad Sci U S A 106: 599–604. 19116272 10.1073/pnas.0804061106 PMC2626749

[30] Tuch BB, Mitrovich QM, Homann OR, Hernday AD, Monighetti CK, et al (2010) The transcriptomes of two heritable cell types illuminate the circuit governing their differentiation. PLoS Genet 6: e1001070. 20808890 10.1371/journal.pgen.1001070 PMC2924316

[31] Eisen MB, Spellman PT, Brown PO, Botstein D (1998) Cluster analysis and display of genome-wide expression patterns. Proc Natl Acad Sci U S A 95: 14863–14868. 9843981 10.1073/pnas.95.25.14863 PMC24541

[32] Tusher VG, Tibshirani R, Chu G (2001) Significance analysis of microarrays applied to the ionizing radiation response. Proc Natl Acad Sci U S A 98: 5116–5121. 11309499 10.1073/pnas.091062498 PMC33173

[33] Huang G, Wang H, Chou S, Nie X, Chen J, et al (2006) Bistable expression of *WOR1*, a master regulator of white-opaque switching in *Candida albicans* . Proc Natl Acad Sci U S A 103: 12813–12818. 16905649 10.1073/pnas.0605270103 PMC1540355

[34] Srikantha T, Borneman AR, Daniels KJ, Pujol C, Wu W, et al (2006) *TOS9* regulates white-opaque switching in *Candida albicans* . Eukaryot Cell 5: 1674–1687. 16950924 10.1128/EC.00252-06 PMC1595353

[35] Zordan RE, Galgoczy DJ, Johnson AD (2006) Epigenetic properties of white-opaque switching in *Candida albicans* are based on a self-sustaining transcriptional feedback loop. Proc Natl Acad Sci U S A 103: 12807–12812. 16899543 10.1073/pnas.0605138103 PMC1535343

[36] Kadosh D, Johnson AD (2005) Induction of the *Candida albicans* filamentous growth program by relief of transcriptional repression: a genome-wide analysis. Mol Biol Cell 16: 2903–2912. 15814840 10.1091/mbc.E05-01-0073 PMC1142434

[37] Simonetti N, Strippoli V, Cassone A (1974) Yeast-mycelial conversion induced by N-acetyl-D-glucosamine in *Candida albicans* . Nature 250: 344–346. 4605454 10.1038/250344a0

[38] Biswas S, Van Dijck P, Datta A (2007) Environmental sensing and signal transduction pathways regulating morphopathogenic determinants of *Candida albicans* . Microbiol Mol Biol Rev 71: 348–376. 17554048 10.1128/MMBR.00009-06 PMC1899878

[39] Sabie F, Gadd GM (1988) Induction of germ-tube formation by *Candida albicans* in amino acid liquid synthetic medium at 25°C. Mycopathologia 101: 77–83. 3278238 10.1007/BF00452890

[40] Doedt T, Krishnamurthy S, Bockmuhl DP, Tebarth B, Stempel C, et al (2004) APSES proteins regulate morphogenesis and metabolism in *Candida albicans* . Mol Biol Cell 15: 3167–3180. 15218092 10.1091/10.1091/mbc.E03-11-0782 PMC452574

[41] Braun BR, Johnson AD (2000) *TUP1*, *CPH1* and *EFG1* make independent contributions to filamentation in *Candida albicans* . Genetics 155: 57–67. 10790384 10.1093/genetics/155.1.57 PMC1461068

[42] Sonneborn A, Tebarth B, Ernst JF (1999) Control of white-opaque phenotypic switching in *Candida albicans* by the Efg1p morphogenetic regulator. Infect Immun 67: 4655–4660. 10456912 10.1128/iai.67.9.4655-4660.1999 PMC96790

[43] Srikantha T, Tsai LK, Daniels K, Soll DR (2000) *EFG1* null mutants of *Candida albicans* switch but cannot express the complete phenotype of white-phase budding cells. J Bacteriol 182: 1580–1591. 10692363 10.1128/jb.182.6.1580-1591.2000 PMC94455

[44] Zordan RE, Miller MG, Galgoczy DJ, Tuch BB, Johnson AD (2007) Interlocking Transcriptional Feedback Loops Control White-Opaque Switching in *Candida albicans* . PLoS Biol 5: e256. 17880264 10.1371/journal.pbio.0050256 PMC1976629

[45] Nie X, Liu X, Wang H, Chen J (2010) Deletion of *EFG1* promotes *Candida albicans* opaque formation responding to pH via Rim101. Acta Biochim Biophys Sin (Shanghai) 42: 735–744. 20870932 10.1093/abbs/gmq076

[46] Braun BR, Johnson AD (1997) Control of filament formation in *Candida albicans* by the transcriptional repressor *TUP1* . Science 277: 105–109. 9204892 10.1126/science.277.5322.105

[47] Braun BR, Kadosh D, Johnson AD (2001) *NRG1*, a repressor of filamentous growth in *C.albicans*, is down- regulated during filament induction. Embo J 20: 4753–4761. 11532939 10.1093/emboj/20.17.4753 PMC125265

[48] Kadosh D, Johnson AD (2001) Rfg1, a protein related to the *Saccharomyces cerevisiae* hypoxic regulator Rox1, controls filamentous growth and virulence in *Candida albicans* . Mol Cell Biol 21: 2496–2505. 11259598 10.1128/MCB.21.7.2496-2505.2001 PMC86882

[49] Murad AM, Leng P, Straffon M, Wishart J, Macaskill S, et al (2001) *NRG1* represses yeast-hypha morphogenesis and hypha-specific gene expression in *Candida albicans* . Embo J 20: 4742–4752. 11532938 10.1093/emboj/20.17.4742 PMC125592

[50] Cleary IA, Mulabagal P, Reinhard SM, Yadev NP, Murdoch C, et al (2010) Pseudohyphal regulation by the transcription factor Rfg1p in *Candida albicans* . Eukaryot Cell 9: 1363–1373. 20656914 10.1128/EC.00088-10 PMC2937334

[51] Park YN, Morschhauser J (2005) Candida albicans *MTLalpha tup1* mutants can reversibly switch to mating-competent, filamentous growth forms. Mol Microbiol 58: 1288–1302. 16313617 10.1111/j.1365-2958.2005.04898.x

[52] Zhao R, Lockhart SR, Daniels K, Soll DR (2002) Roles of *TUP1* in switching, phase maintenance, and phase-specific gene expression in *Candida albicans* . Eukaryot Cell 1: 353–365. 12455984 10.1128/EC.1.3.353-365.2002 PMC118011

[53] Carlisle PL, Kadosh D (2010) *Candida albicans* Ume6, a filament-specific transcriptional regulator, directs hyphal growth via a pathway involving Hgc1 cyclin-related protein. Eukaryot Cell 9: 1320–1328. 20656912 10.1128/EC.00046-10 PMC2937344

[54] Zheng XD, Lee RT, Wang YM, Lin QS, Wang Y (2007) Phosphorylation of Rga2, a Cdc42 GAP, by CDK/Hgc1 is crucial for *Candida albicans* hyphal growth. EMBO J 26: 3760–3769. 17673907 10.1038/sj.emboj.7601814 PMC1952229

[55] Zeidler U, Lettner T, Lassnig C, Muller M, Lajko R, et al (2009) *UME6* is a crucial downstream target of other transcriptional regulators of true hyphal development in *Candida albicans* . FEMS Yeast Res 9: 126–142. 19054126 10.1111/j.1567-1364.2008.00459.x

[56] Feng Q, Summers E, Guo B, Fink G (1999) Ras signaling is required for serum-induced hyphal differentiation in *Candida albicans* . J Bacteriol 181: 6339–6346. 10515923 10.1128/jb.181.20.6339-6346.1999 PMC103768

[57] Lane S, Birse C, Zhou S, Matson R, Liu H (2001) DNA array studies demonstrate convergent regulation of virulence factors by Cph1, Cph2, and Efg1 in *Candida albicans* . J Biol Chem 276: 48988–48996. 11595734 10.1074/jbc.M104484200

[58] Schweizer A, Rupp S, Taylor BN, Rollinghoff M, Schroppel K (2000) The TEA/ATTS transcription factor CaTec1p regulates hyphal development and virulence in *Candida albicans* . Mol Microbiol 38: 435–445. 11069668 10.1046/j.1365-2958.2000.02132.x

[59] Brown DH Jr, Giusani AD, Chen X, Kumamoto CA (1999) Filamentous growth of *Candida albicans* in response to physical environmental cues and its regulation by the unique *CZF1* gene. Mol Microbiol 34: 651–662. 10564506 10.1046/j.1365-2958.1999.01619.x

[60] Nantel A, Dignard D, Bachewich C, Harcus D, Marcil A, et al (2002) Transcription profiling of *Candida albicans* cells undergoing the yeast-to-hyphal transition. Mol Biol Cell 13: 3452–3465. 12388749 10.1091/mbc.E02-05-0272 PMC129958

[61] Zhu W, Filler SG (2009) Interactions of *Candida albicans* with epithelial cells. Cell Microbiol 12: 273–282. 19919567 10.1111/j.1462-5822.2009.01412.x PMC3383095

[62] Nobile CJ, Andes DR, Nett JE, Smith FJ, Yue F, et al (2006) Critical role of Bcr1-dependent adhesins in *C. albicans* biofilm formation in vitro and in vivo. PLoS Pathog 2: e63. 16839200 10.1371/journal.ppat.0020063 PMC1487173

[63] Wolyniak MJ, Sundstrom P (2007) Role of actin cytoskeletal dynamics in activation of the cyclic AMP pathway and *HWP1* gene expression in *Candida albicans* . Eukaryot Cell 6: 1824–1840. 17715368 10.1128/EC.00188-07 PMC2043390

[64] Crampin H, Finley K, Gerami-Nejad M, Court H, Gale C, et al (2005) *Candida albicans* hyphae have a Spitzenkorper that is distinct from the polarisome found in yeast and pseudohyphae. J Cell Sci 118: 2935–2947. 15976451 10.1242/jcs.02414

[65] Liu H (2002) Co-regulation of pathogenesis with dimorphism and phenotypic switching in *Candida albicans*, a commensal and a pathogen. Int J Med Microbiol 292: 299–311. 12452278 10.1078/1438-4221-00215

[66] Lachke SA, Lockhart SR, Daniels KJ, Soll DR (2003) Skin facilitates *Candida albicans* mating. Infect Immun 71: 4970–4976. 12933839 10.1128/IAI.71.9.4970-4976.2003 PMC187354

[67] Bennett RJ, Johnson AD (2006) The role of nutrient regulation and the Gpa2 protein in the mating pheromone response of *C. albicans* . Mol Microbiol 62: 100–119. 16987174 10.1111/j.1365-2958.2006.05367.x

[68] Banerjee M, Uppuluri P, Zhao XR, Carlisle PL, Vipulanandan G, et al (2012) Expression of *UME6*, a key regulator of *C. albicans* hyphal development, enhances biofilm formation via Hgc1- and Sun41-dependent mechanisms. Eukaryot Cell 12: 224–232. 23223035 10.1128/EC.00163-12 PMC3571304

