# Supplementary figures and images for: Candida albicans White and Opaque Cells Undergo Distinct Programs of Filamentous Growth
Source: PLoS Pathog. 2013 Mar 7;9(3):e1003210. doi: 10.1371/journal.ppat.1003210 (PMC3591317; doi:10.1371/journal.ppat.1003210)

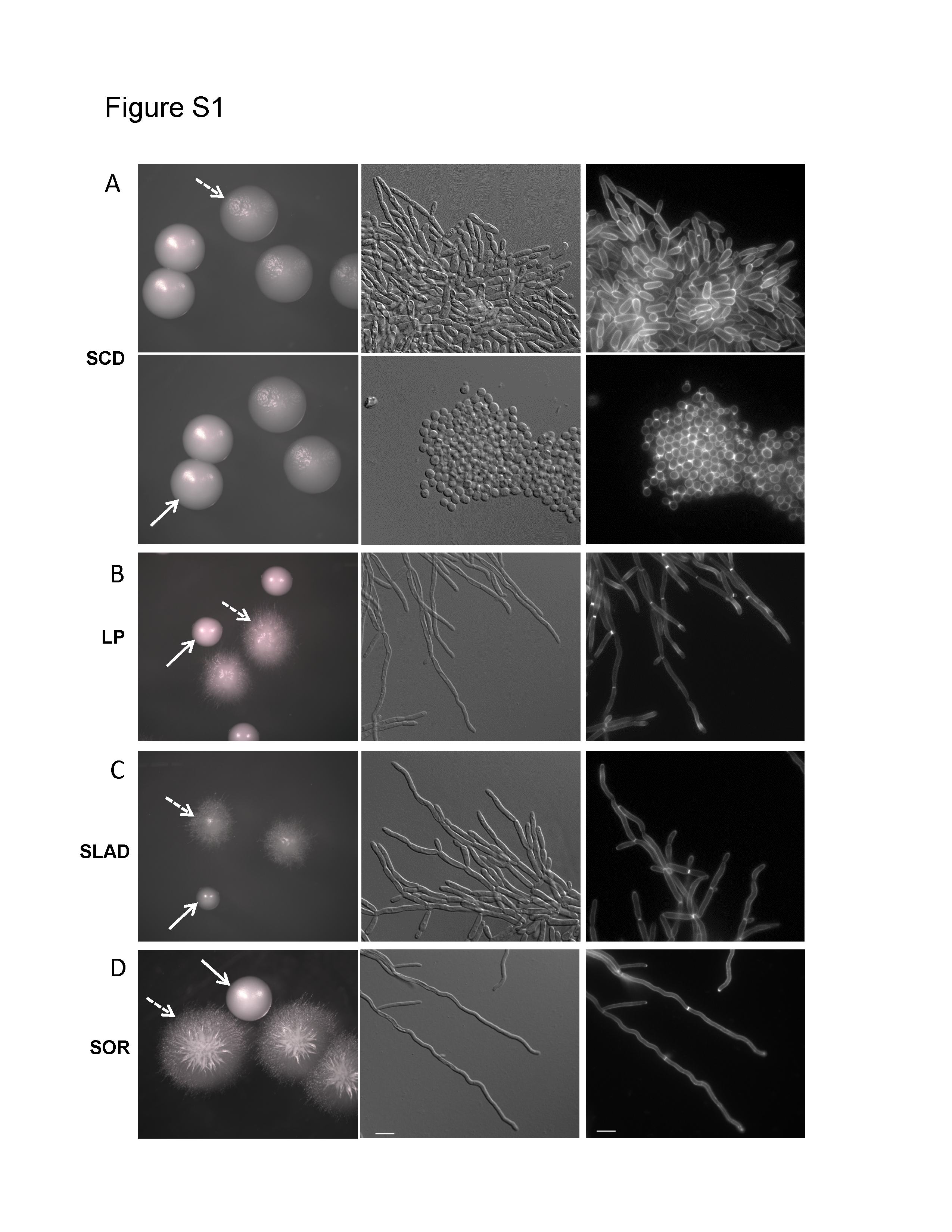

Supplement: Figure S1 — Natural opaque cells undergo filamentation in response to environmental cues. Culture conditions induce filamentous growth in wildtype opaque cells as well as in opaque-locked cells (see Figure 2). Strain RBY731 (opaque form of strain RBY717) was grown on (A) SCD (B) low phosphate (LP), (C) low nitrogen (SLAD), or (D) sorbitol (SOR) medium at 25°C. These culture conditions do not induce filamentation in white cells. Panels show colony morphologies from mixed white/opaque populations (solid arrow, white colonies; dashed arrow, opaque colonies) after 4 days growth. Additional panels show DIC images of white and opaque cells, as well as calcofluor white (CW)-stained cells after 22 hours growth. Scale bar, 10 µm. (TIFF) [file ppat.1003210.s001.tif]

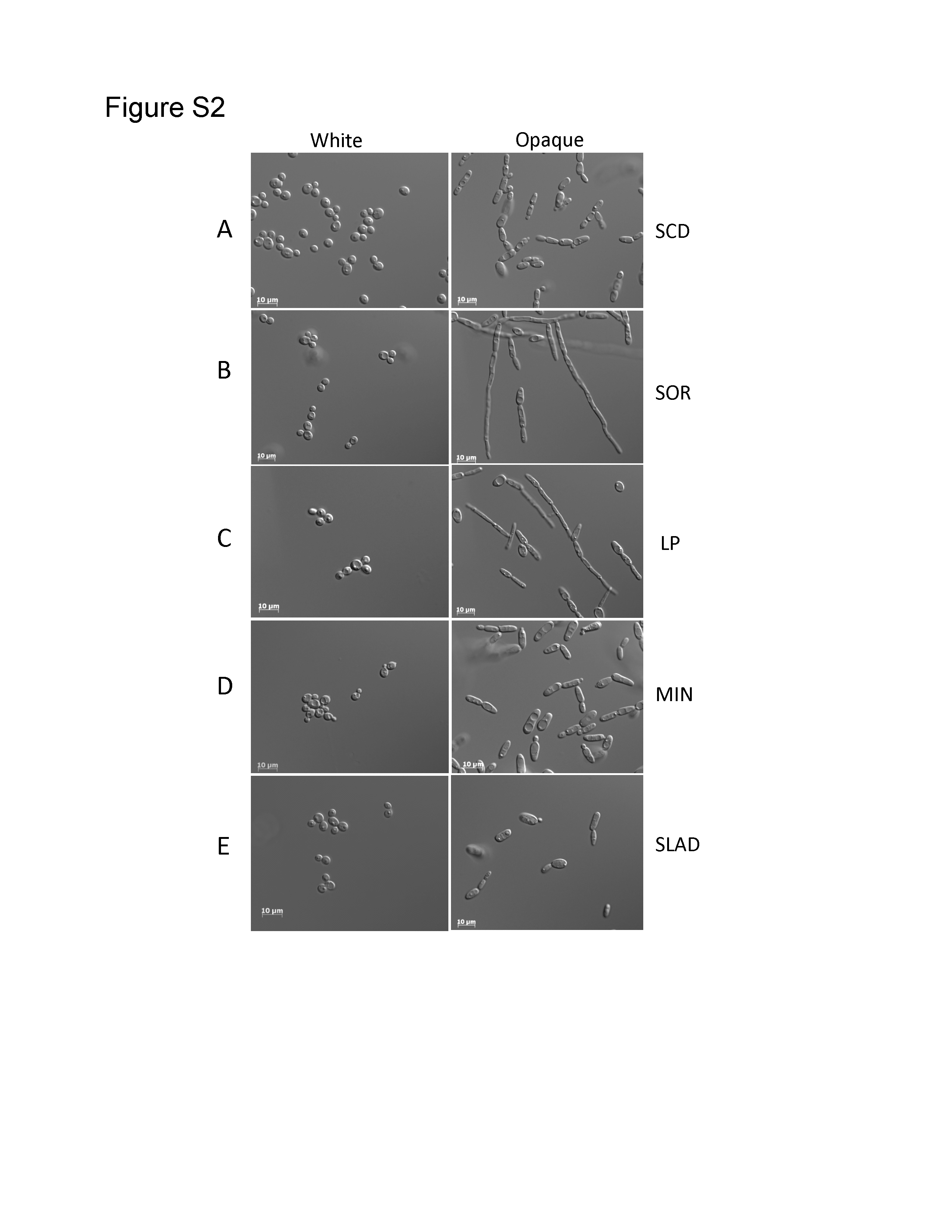

Supplement: Figure S2 — Opaque cells undergo filamentation in liquid culture media. Opaque cells (strain CAY2903) undergo filamentation when cultured in liquid media, in addition to growth on solid media (Figure 2). Images were taken after 16 hours incubation at 25°C. (A), SCD, (B), SOR, (C), LP, (D), MIN, and (E) SLAD medium. Opaque cell filamentation is strongest in liquid SOR and LP media. (TIF) [file ppat.1003210.s002.tif]

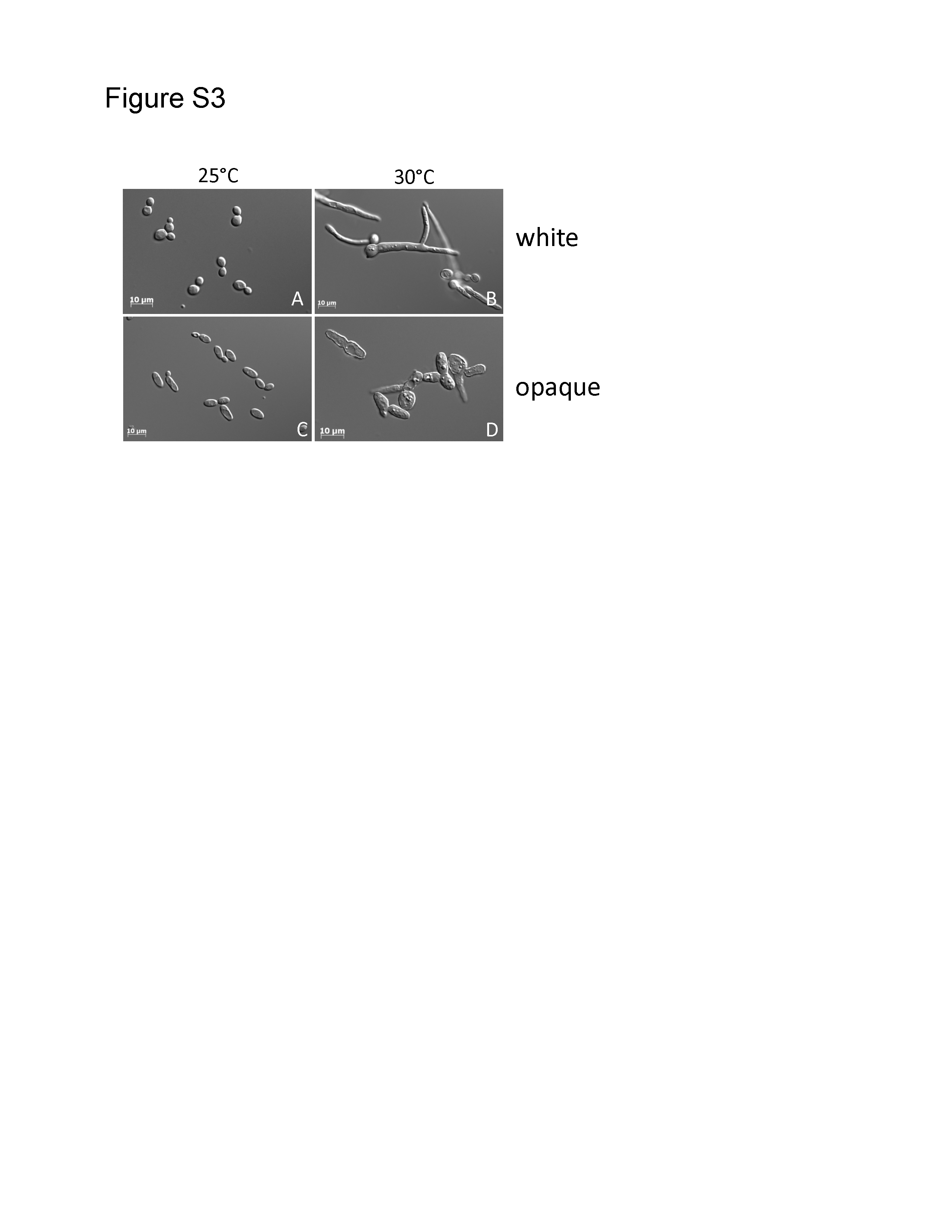

Supplement: Figure S3 — Contrasting Hsp90-mediated regulation of morphogenesis in white and opaque cells. White cells (RBY717) treated with the Hsp90 inhibitor geldanamycin (GdA) were induced to undergo filamentous growth at 30°C, but not 25°C. In contrast, opaque cells (CAY2903) did not undergo efficient filamentation when incubated with GdA at either temperature. Cells were grown at 25°C (A and C) or 30°C (B and D) and treated with 10 µM GdA for 12 hours. (TIFF) [file ppat.1003210.s003.tif]

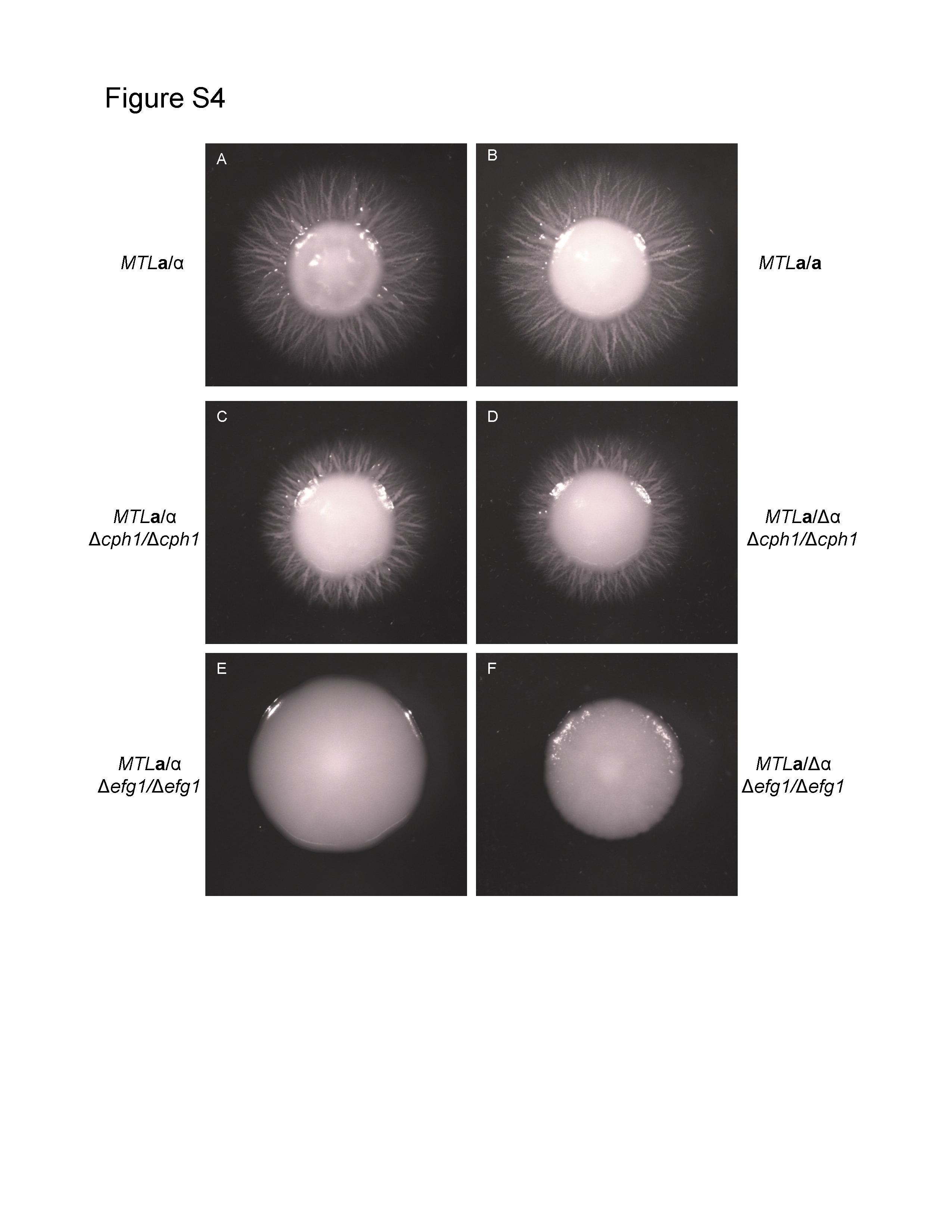

Supplement: Figure S4 — Comparison of cph1 and efg1 mutant phenotypes in white MTL a/α and MTL a strains. All strains were grown on Spider medium at 30°C for 4 days and photographed. (A) Wildtype white a/α, (B) wildtype white a/a strain (RBY717), (C) cph1 white a/α strain (CJN2741), (D) cph1 white a/Δα strain (CAY4479), (E) efg1 white a/α strain (CAY4522), (F) efg1 white a/Δα strain (CAY3526). In both MTL heterozygous and MTL homozygous strains the cph1 mutant had a subtle defect in filamentation while the efg1 mutant had a marked defect in filamentation. (TIF) [file ppat.1003210.s004.tif]

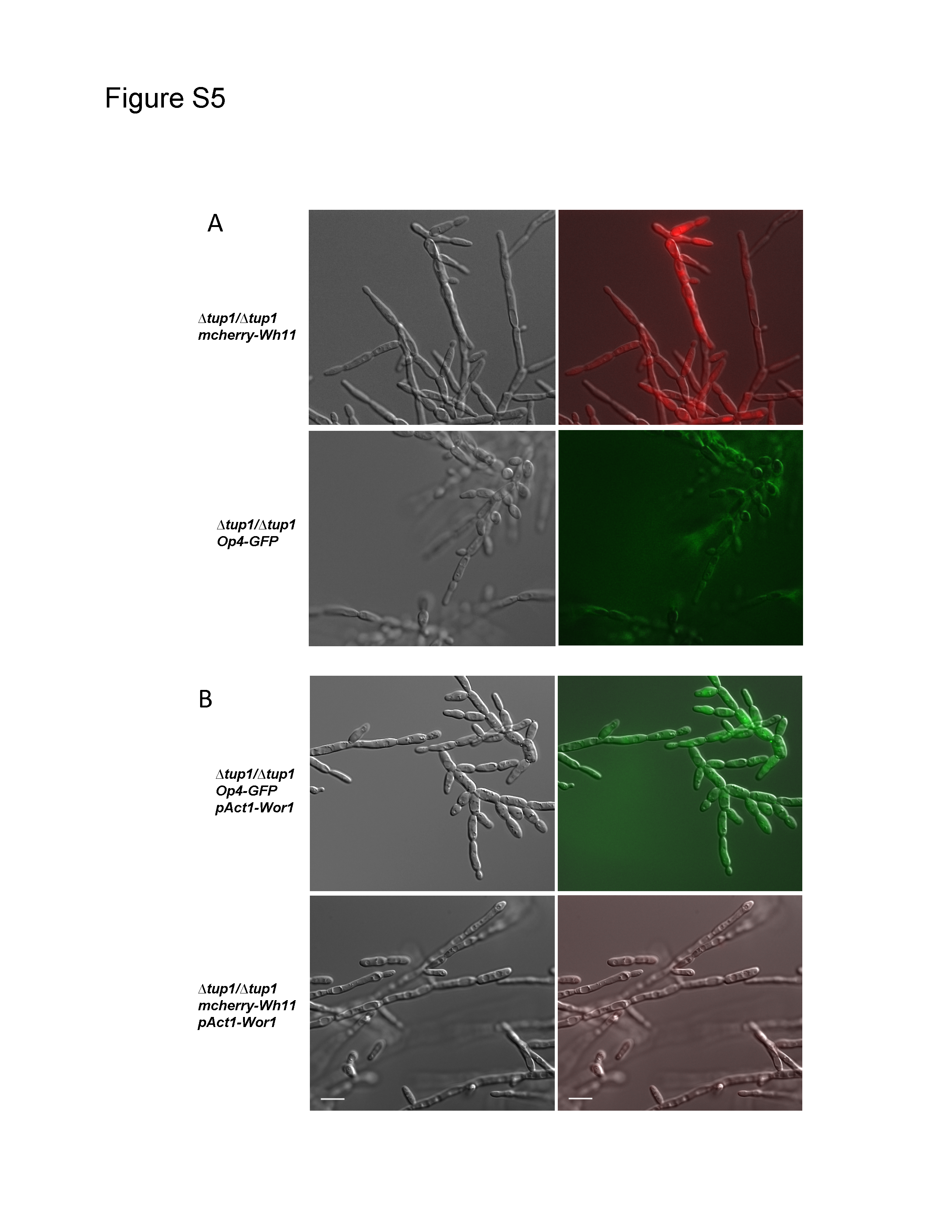

Supplement: Figure S5 — Deletion of TUP1 induces filamentous growth in both white and opaque cells. The role of the transcription factor Tup1 was tested in both white and opaque programs of filamentous growth using fluorescent reporters to confirm the phenotypic state of the cell. (A) White tup1 mutants expressing a white-specific reporter (pWH11-mCherry) or an opaque-specific reporter (pOP4-GFP) in strains CAY4356 or CAY4353, respectively. Strong expression of the WH11 gene (high mCherry levels) confirms that white tup1 mutants are undergoing filamentation. (B) Opaque tup1 mutants expressing white and opaque reporter constructs in strains CAY4492 and CAY4291. Strong expression of the opaque-specific OP4 reporter confirms that tup1 mutants are propagating in the opaque state and undergoing constitutive filamentous growth similar to that of white cells. Cells were grown for 16 h in SCD medium and photographed. Scale bar, 10 µm. (TIF) [file ppat.1003210.s005.tif]

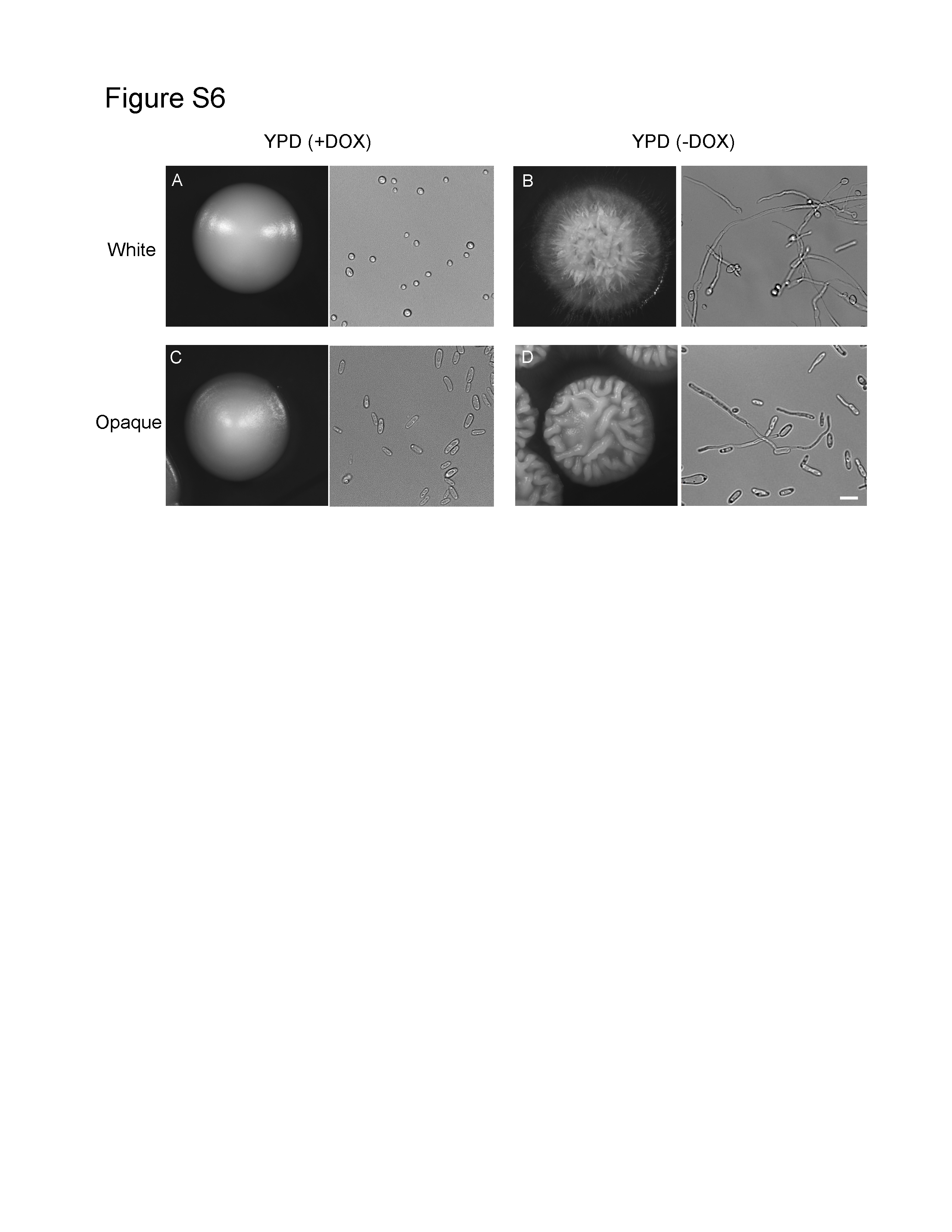

Supplement: Figure S6 — Induction of UME6 expression induces filamentation in both white and opaque cells. The UME6 gene was placed under the control of the tetO operator in a strain expressing the E. coli tet repressor - S. cerevisiae Hap4 activation domain fusion protein. In the presence of Dox (doxycycline) the UME6 gene is repressed (A and C), while in the absence of Dox the UME6 gene is induced (B and D). In both white (CAY4504) and opaque (CAY4502) cells filamentous growth occurred when grown on YPD without doxycycline. Colonies were grown for 6 days at 25°C. Scale bar, 10 µm. (TIF) [file ppat.1003210.s006.tif]

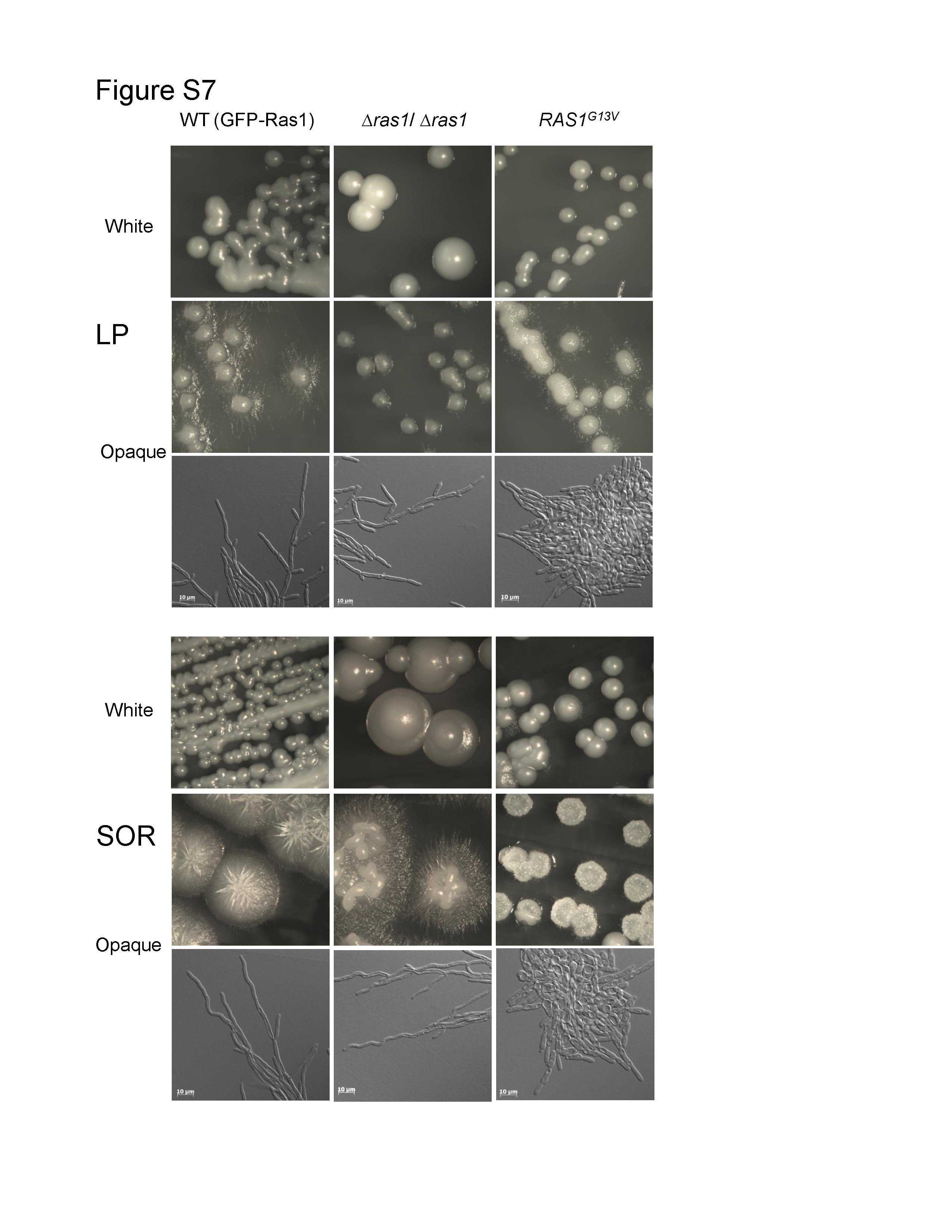

Supplement: Figure S7 — Deletion or overexpression of RAS1 leads to a defect in opaque filamentation. Wildtype cells (expressing a Ras1-GFP fusion protein), Δras1/Δras1 mutants, or cells expressing a constitutively active Ras1 allele (G13V) were compared for their ability to undergo filamentation in the opaque state. Both ras1 mutants and strains expressing hyperactive RAS1 alleles showed decreased filamentation on LP and SOR medium relative to the control strain. Strains were incubated on media for 4 days at 25°C. Strains used were wildtype white cells (CAY3749), opaque cells (CAY3619), ras1 white cells (CAY2723), ras1 opaque cells (CAY2795), and constitutively active Ras1 white cells (CAY3751) and opaque cells (CAY3621). (TIF) [file ppat.1003210.s007.tif]

Figure S8A

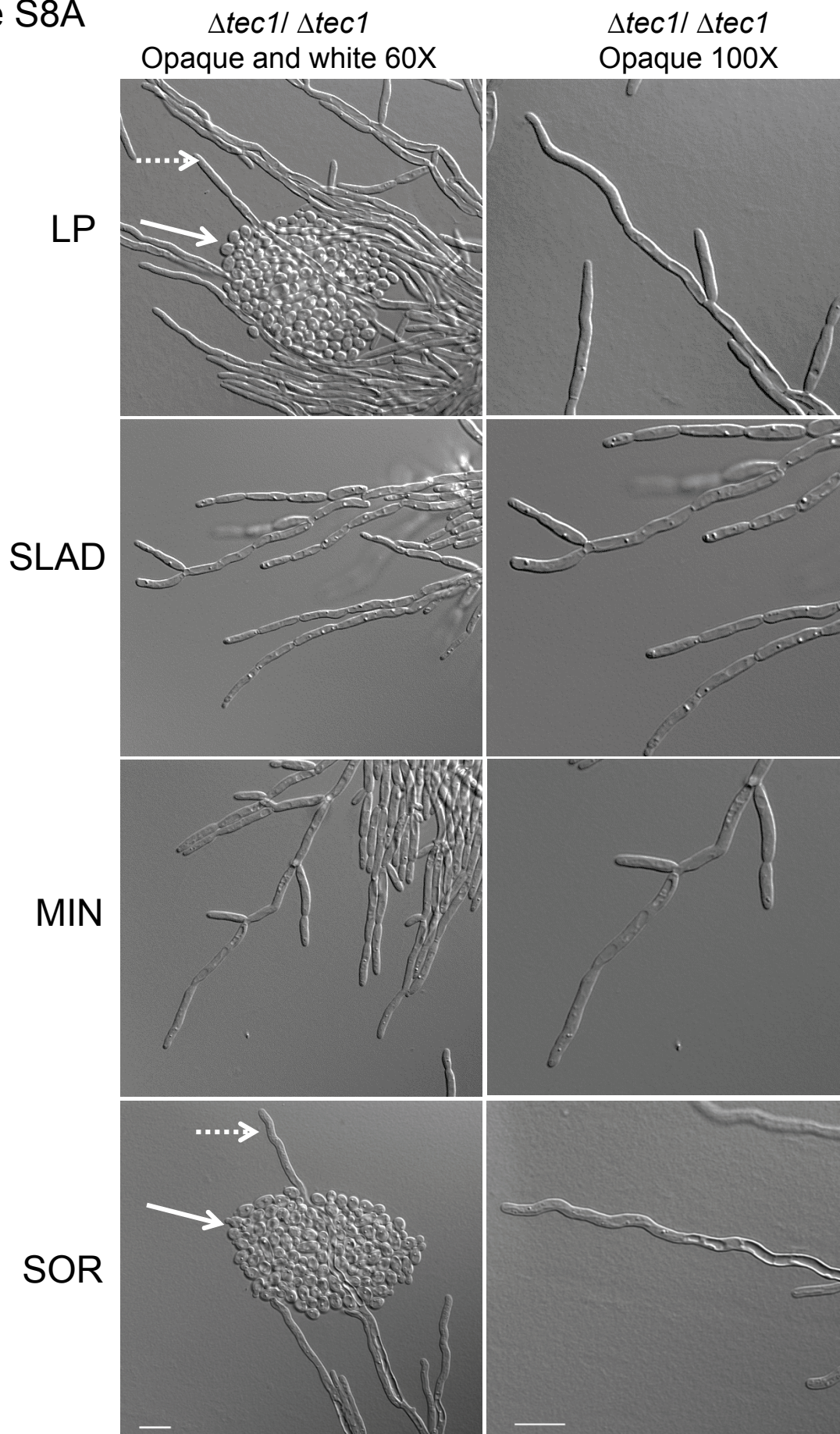

Figure S8B

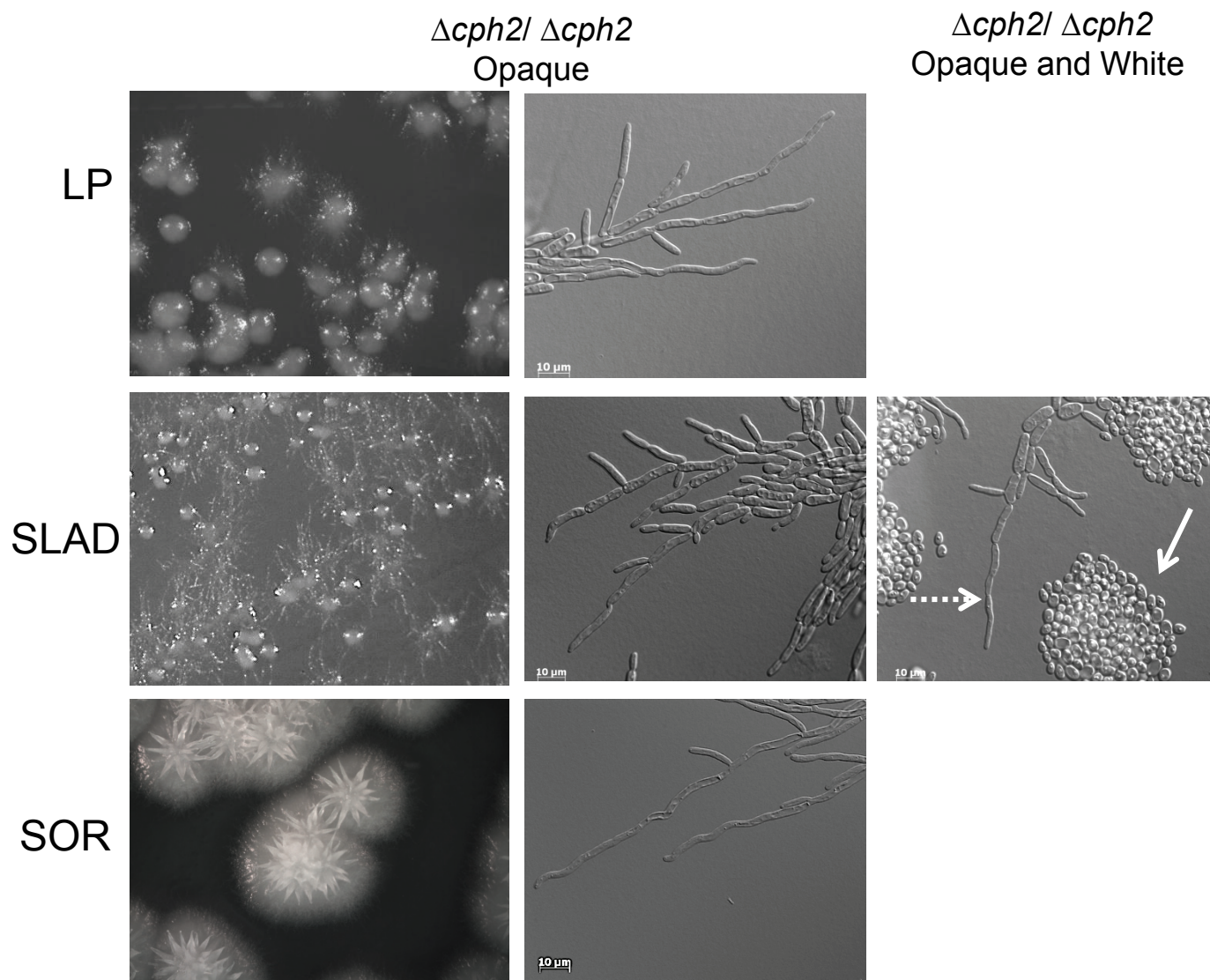

Supplement: Figure S8 — Analysis of the role of Cph2 and Tec1 in opaque filamentation. Mutants lacking (A) Tec1 and (B) Cph2 were analyzed for opaque filamentation phenotypes. Neither of these factors appeared to play a significant role in filamentous growth in opaque cells. Strains were incubated on media for 22 hours (cell images) or 4 days (colony images) at 25°C. Cph2 mutants used were CAY2091 (white cells) and CAY3296 (opaque cells). Tec1 mutants used were CAY2646 (white cells) and CAY2688 (opaque cells). Solid arrow, white cells; dashed arrow, opaque cells. Scale bar, 10 µm. (PDF) [file ppat.1003210.s008.pdf]

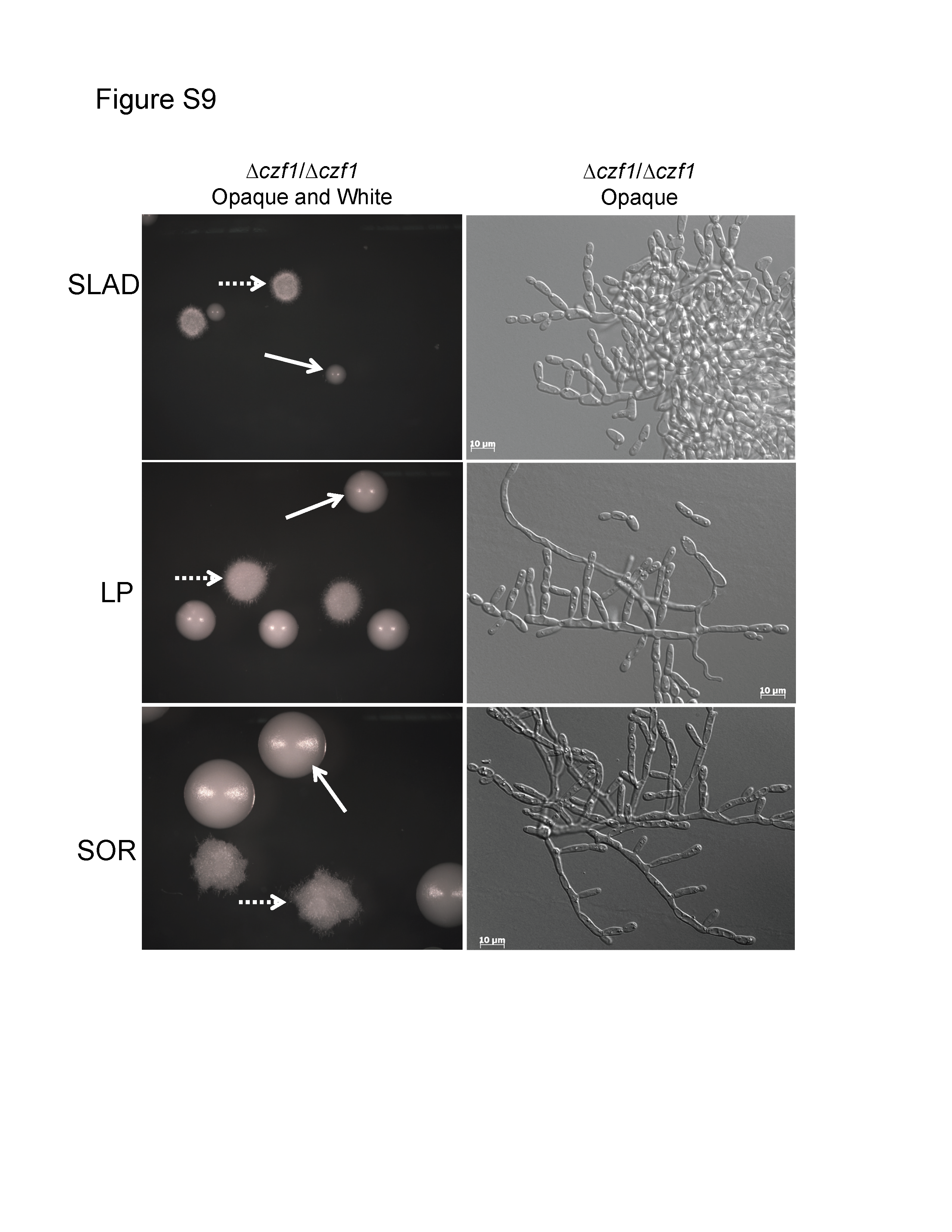

Supplement: Figure S9 — Analysis of the role of Czf1 in opaque filamentation. Opaque cells (CAY3294) lacking the transcription factor Czf1 were found to exhibit a hyper-branching phenotype when grown on LP, SLAD and SOR medium. In contrast, white czf1 mutants (CAY3522) did not exhibit this phenotype. Solid arrow, white colonies; dashed arrow, opaque colonies. Cells were imaged after 22 h and colonies after 4 d at 25°C. (TIF) [file ppat.1003210.s009.tif]

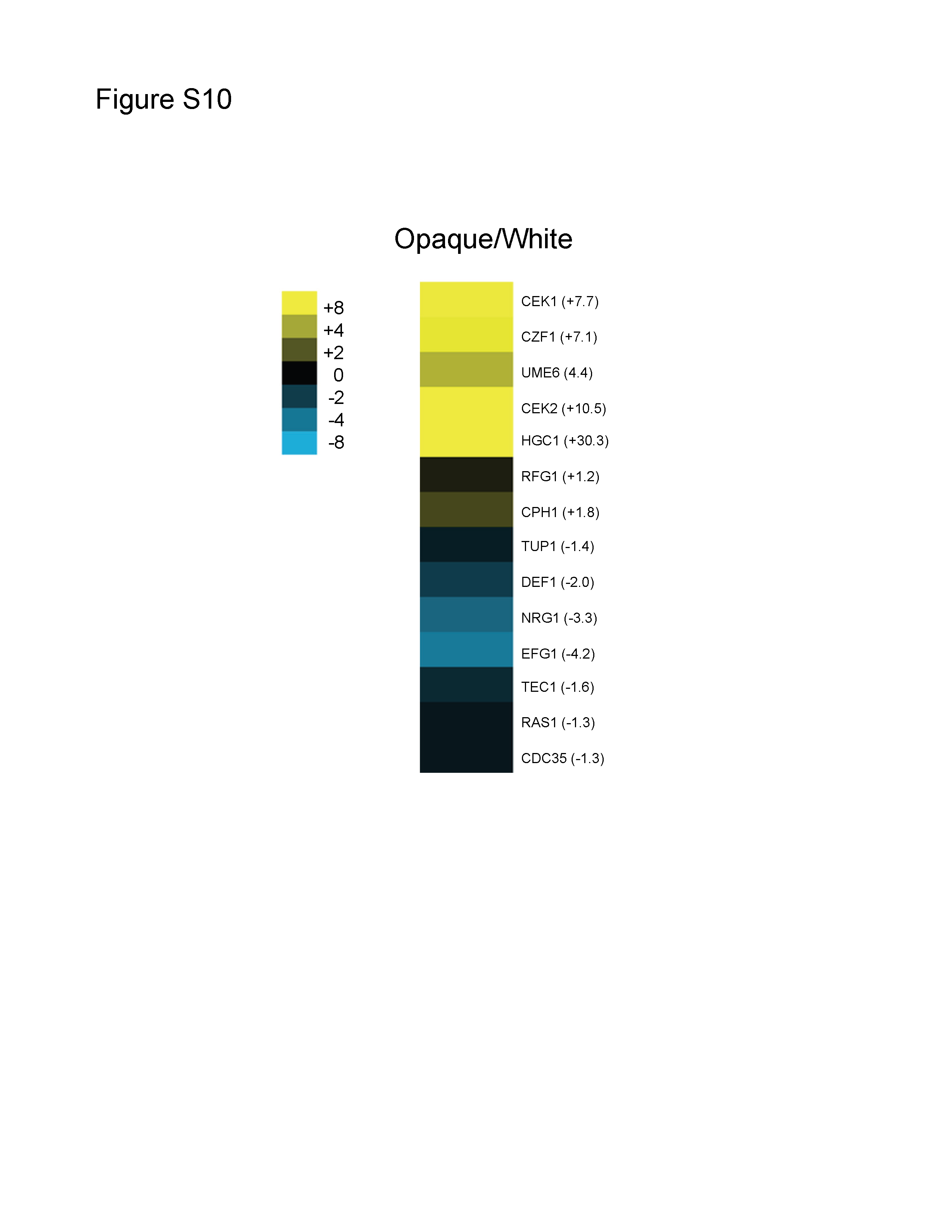

Supplement: Figure S10 — Comparative expression of yeast-hyphal regulators between white and opaque cells. Gene expression was compared for multiple yeast-hyphal regulators between white and opaque cells and revealed that many of these regulators are expressed in a phase-specific pattern. For example, the regulators CZF1, UME6, HGC1, CEK1, and CEK2 are expressed at an elevated level (>4-fold) in opaque cells compared to white cells. In contrast, other established yeast-hyphal regulators including EFG1 and NRG1 are expressed more in white cells than opaque cells (>3-fold). Finally some regulators are not differentially expressed between white and opaque cells (e.g. CPH1 and RFG1). Fold change in expression is shown in parentheses. Data adapted from RNA-seq analysis in Tuch et al. [30]. (TIF) [file ppat.1003210.s010.tif]
