# Supplementary material for: Candida albicans White and Opaque Cells Undergo Distinct Programs of Filamentous Growth
Source: PLoS Pathog. 2013 Mar 7;9(3):e1003210. doi: 10.1371/journal.ppat.1003210 (PMC3591317; doi:10.1371/journal.ppat.1003210)
Supplement: Table S1 — Oligonucleotides used in this study. (DOCX) [file ppat.1003210.s011.docx]

**Table S1**. Primers used in this study.

| **OLIGO** | **NAME** | **SEQUENCE** |
| --- | --- | --- |
| 127 | ARG4 LEFT | TTCAACCTTTCAAACGATGC |
| 128 | ARG4 RIGHT | TCGATACATTTGCGGTACAG |
| 169 | SAT1FOR XHOI | GgcgccCTCGAGtgattcatcccattcattcc |
| 170 | SAT1REV XBAI | GgcgcgTCTAGAgtctgcaggaccacctttga |
| 225 | LEU2 LEFT | AGAATTCCCAACTTTGTCTG |
| 226 | LEU2 RIGHT | AAACTTTGAACCCGGCTGCG |
| 610 | ACT1(-950)APAI | GGAGCGGGGCCCGGACCAAAATAGAAACTGTTCTCAGG |
| 611 | ACT1(0) | GAATGATTATATATTTTTTTAATATTAATATCGAGATAATG |
| 690 | HIS1 LEFT | ATTAGATACGTTGGTGGTTC |
| 691 | HIS1 RIGHT | AACACAACTGCACAATCTGG |
| 982 | CPH1UPREV | CACGGCGCGCCTAGCAGCGG  TGGCGAAAGAGTGTATTAGGCAG |
| 983 | CPH1DNFOR | GTCAGCGGCCGCATCCCTGC CAGAGAGAAAGAGAAAGAAAG |
| 984 | RFG1UPREV | CACGGCGCGCCTAGCAGCGGTTTGTTTATTTGGTGGGTTGCC |
| 985 | RFG1DNFOR | GTCAGCGGCCGCATCCCTGCTGCTGCTCAACATTATCAACA |
| 990 | CPH1UPFOR | TTTTGTTGTTCCCCGTTACCC |
| 991 | CPH1DNREV | GAATGTGTTCATTCAATAAAGATAG |
| 992 | RFG1UPFOR | TTCCTTTGGTGACTCCGATTTTC |
| 993 | RFG1DNREV | GGATTAACCCTTTTTCTCGTCA |
| 1026 | RFG1ORF5' | CCTCCACCACCACATTCTCAAC |
| 1027 | RFG1ORF3' | CACCACCACTACCACTCATTCCAC |
| 1215 | EFG1UPREV | CACGGCGCGCCTAGCAGCGGGCAGTTGTTTGCTGGGGCATA |
| 1216 | EFG1 DWFOR | GTCAGCGGCCGCATCCCTGCTGACTCAAGGTTCAGTTCACC |
| 1217 | EFG1 DWREV | CCTTCCGCATTAGACGCTTA |
| 1254 | EFG1CHE LEFT2 | TTGCCCTACCCATCTACTCG |
| 1255 | EFG1CHE RIGHT2 | CATCACCTGTAACTCGTGTCG |
| 1384 | WH11-PROM (-900) | GGCGCCGGGCCCCTGAAACACCATTGTTGATTGC |
| 1385 | FP-FOR | CGGATCCCCGGGTTAATTAACGG |
| 1386 | FP-REV | GGCCGCGTCGACCAACTCAGAGCACGCTAGAC |
| 1387 | WH11-PROM CHECK (-950) | GTCGTTATTGAAGGCTACTTG |
| 1389 | OP4-PROM CHECK (-900) | GCATGTAACCACAATCAAAACC |
| 1396 | WH11-PROM (0) | CCGTTAATTAACCCGGGGATCCGGTTTAATTGTTCTGTTTGTTGTTTTG |
| 1473 | GFP REV (+300) | AACCTTCTGGCATGGCAGAC |
| 1474 | MCHERRY REV +300 | CAA GTA GTC TGG GAT ATC AGC |
| 1651 | PYM70 PSTI | GGCGCCCTGCAGCATGATAATAATGGTTTCTTAGACGTC |
| 1652 | PSFS2 REV | CACGGCGCGCCTAGCAGCGGATAGAAATGCCTTGGGTGGC |
| 1653 | PYM70 | CCGCTGCTAGGCGCGCCGTGCCACCTGACGTCGTATAGTG |
| 1669 | PSFS2 FOR | CTTAGTCAGATCGTACAATAAAGC |
| 1829 | CZF1ABF | GGCCGCGGGCCCTAGAATCTTTTACCCGAACAGC |
| 1830 | CZF1ABR | CCGCGCCTCGAGTTGTAGAACTCAAATACCCTCTCAC |
| 1831 | CZF1ABCHE | TCCCCTACACATTTGGACTACCC |
| 1832 | UME6ABF | GGCCGCGGGCCCCCAGAAAACTCATAAACTTCTCCG |
| 1833 | UME6ABR | CCGCGCCTCGAGTGGAATCTTCTACGGGAAAAG |
| 1834 | UME6ABCHE | CGCATAAACAAGCACCAAACACC |
| 1837 | RFG1ABCHE | TTCACCACCCCTCATCATTCC |
| 1838 | EFG1ABF | CGGCCGGGTACCATTGCCCTACCCATCTACTCGC |
| 1839 | EFG1ABR | GGCCGCGGGCCCTGTCAATGGATTTGGGAGAAG |
| 1840 | EFG1ABCHE | GGGGAAGCAAAACTAAGAAAAGTAG |
| 1846 | MCH-1 FOR | GGCGCCGGATCCCGGATCCCCGGGTTAATTAACGG |
| 1847 | MCH-1 REV | GCCGGCCTTAAGCTTGTACAATTCATCCATACCACC |
| 1848 | MCH-2 FOR | GGCGCCCTTAAGATGGTTTCTAAGGGTGAAGAAG |
| 1849 | MCH-2 REV | GCCGGCAGGCCTCTTGTACAATTCATCCATACCACC |
| 1850 | MCH-3 FOR | GGCGCCAGGCCTATGGTTTCTAAGGGTGAAGAAG |
| 1974 | OP4 KPNI | GGCGCCGGTACCCCGACATTTATTGAGGGAAC |
| 1975 | OP4 SACI | GCCGGCGAGCTCTGTAAATTATTTATATTTGTATGTGTGTAGG |
| 2421 | WOR1(0)FUSION | CATTATCTCGATATTAATATTAAAAAAATATAATCATTCAAAATGTCTAATTCAAGTATAGTCCCTAC |
| 2422 | WOR1 XHOI (+2600) | GCCGGCCTCGAGCATCTAAACCTATAGTACAACAC |
| 2423 | CHECPH1UPF | TTGTTTCCCCATTGACACG |
| 2424 | CHECPH1DWR | TATACGCAGCTTAGATGGGGTTG |
| 2425 | EFG1UPFOR | ACATCCAGCCAACCCACTTA |
| 2426 | CHERFG1UPF | CAAGGATTTTCACACACCTGCAC |
| 2427 | CHERFG1DWR | ATTGCTCAAGCGTGCACACACC |
| 2428 | UME6 FOR | CCTCAACAACAAGCACAATCCC |
| 2429 | UME6 REV | TTGGTGGTGGTGGAAGAGAAGG |
| 2430 | QHGC1FOR1 | CGATACCACCACCACAAATGC |
| 2431 | QHGC1 REV1 | TGATGAGGTGCAGGAAGCTGAC |
| 2432 | QPAT1 F1 | CCAAGAAAGCCCAGAAAGTGG |
| 2433 | QPAT1 R1 | CTGCTGTTGTTGTTGTTGTGGAGC |
| 2490 | CaLeu2F | TTTCCACACTGATGCTGCTC |
| 2491 | CaLeu2R | TAACTACCTCGGCACCTTCC |
| 2492 | CaHis1F | GCATGGATTGTTGTGTGAGG |
| 2493 | CaHis1R | CAGCCCATTGAACAAATTGA |
| 2494 | CaArg4F | GACAGGAATCACGTCATCGTT |
| 2495 | CaArg4R | TGGCCCTTGGTATTAGGTGA |
